# Regioselective chlorination and bromination of unprotected anilines under mild conditions using copper halides in ionic liquids

**DOI:** 10.3762/bjoc.8.84

**Published:** 2012-05-16

**Authors:** Han Wang, Kun Wen, Nurbiya Nurahmat, Yan Shao, He Zhang, Chao Wei, Ya Li, Yongjia Shen, Zhihua Sun

**Affiliations:** 1 School of Chemistry and Molecular Engineering, East China University of Science and Technology, Shanghai, 200237, China; 2 College of Chemistry and Chemical Engineering, Shanghai University of Engineering Science, Shanghai, 201620, China

**Keywords:** halogenation, ionic liquids, oxydation, regioselectivity, solvent effects

## Abstract

By using ionic liquids as solvents, the chlorination or bromination of unprotected anilines at the para-position can be achieved in high yields with copper halides under mild conditions, without the need for potentially hazardous operations such as supplementing oxygen or gaseous HCl.

## Introduction

Chlorination and bromination of aromatic rings are classical and widely performed transformations, which are useful in many multistep organic-synthesis procedures. Regioselectivity with electron-rich substrates such as aniline will predominantly produce para- and ortho-substitutions. However, chlorination or bromination of aniline derivatives is often performed with protected anilines. This increases both the cost of synthesis and the environmental impact due to the added protection and deprotection reaction steps.

Direct chlorination of unprotected aniline is possible by using sulfonyl chloride [1], chlorine [2–3] or *N*-chlorosuccinimide [4]. However, from an environmental-impact point of view, probably the best chlorination protocol was the one reported several decades ago with chloride salts of several transition metals [5]. Copper(II) chloride (**1**) was found to be one of the best reagents for this transformation, which yielded mostly the para-chlorinated product with minor ortho- and dichlorinated products. The mechanism was believed to be mediated by Cu(II) oxidation of aniline, followed by addition of the chloride.

Despite the good yield and regioselectivity in the original report on the chlorination of unprotected aniline with CuCl_2_, its application to other unprotected aniline derivatives over the years has not been widely reported. Instead, many procedures still opted for the chlorination of protected aniline derivatives or the introduction of nitrogen (for example, through nitration) to chlorobenzenes. Part of the reason could be that the procedure with CuCl_2_ is carried out in concentrated aqueous HCl and requires the flow of both oxygen gas and HCl gas to achieve high yield. The supplemented oxygen gas reoxidizes the Cu(I) product back to Cu(II) to keep the reaction efficient, while the HCl gas keeps the polymerization of unprotected aniline to a minimum under these conditions. This promoted us to search for conditions of this transformation that do not require the use of aqueous or gaseous HCl and oxygen gas.

**Scheme 1:**
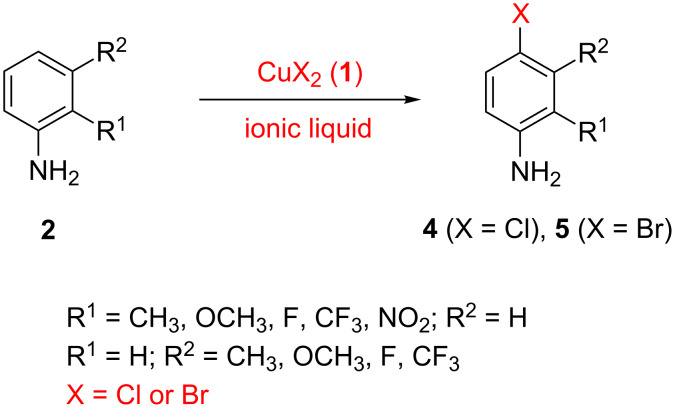
Chlorination (or bromination) of unprotected aniline by CuX_2_.

Our search for suitable conditions focused on the use of ionic liquids as solvents. The amount of research into the application of ionic liquids in organic synthesis has experienced a dramatic expansion over the past decade. This has been driven by the desires to both find green alternatives to conventional solvents, through the physical properties of ionic liquids, and to explore improvements in organic transformations in terms of efficiency and ease of operation [6–11]. In this article, we show that ionic liquids enable the title transformation to be achieved under mild conditions without the need for oxygen or gaseous HCl (Scheme 1).

## Results and Discussion

We first used 2-methylaniline (**2a**) to explore and compare chlorination conditions using CuCl_2_ either in water or in an ionic liquid, with or without the use of either oxygen, gaseous HCl, or both. The results are summarized in Table 1. The choice of 2-methylaniline will leave one position for either para- or ortho-chlorination, thus simplifying the product analysis.

**Table 1:** Exploring solvents and other conditions for the chlorination of 2-methylaniline (**2a**, 10 mmol scale) using CuCl_2_.^a^

entry	solvent	CuCl_2_ equiv	O_2_	HCl (g)	*T* (°C)	time	2-methyl- aniline remaining	4-Cl product **4a**	2-Cl product **4a’**	di-Cl product **4a’’**

1	36% aq HCl	2	Yes	Yes	40	2 d	55%	43%	1%	—
2	36% aq HCl	2	Yes	Yes	60	6 h	10%	82% (75%)^b^	5.8%	1.6%
3	36% aq HCl	3	Yes	Yes	60	6 h	8%	82%	6.5%	2.1%
4	36% aq HCl	2	No	Yes	60	3 h	100%	—	—	—
5	36% aq HCl	2	Yes	No	60	3 h	85%	10%	1.5%	—
6	1-hexyl-3-methyl- imidazolium chloride (**3a**)	3	No	No	40	4 h	5%	92% (91%)^b^	2.5%	—
7	1-isobutyl-3-methyl- imidazolium chloride (**3b**)	3	No	No	40	4 h	7%	89%	3.2%	—
8	1-pentyl-3-methyl- imidazolium chloride (**3c**)	3	No	No	40	4 h	10%	88%	2.8%	—

^a^Product distribution was analyzed by GC–MS. ^b^Isolated yields.

The reactions were carried out on a 10 mmol scale (~1 g) of 2-methylaniline (**2a**). To compare the conversion efficiencies of all trials, we used GC–MS-based analysis of crude reaction samples after normal workup procedures. For a few representative cases, isolated yields were also listed in Table 1.

For reactions in 36% aqueous HCl, the use of oxygen and gaseous HCl was essential to obtain good yields for para-chlorination of 2-methylaniline (Table 1, entries 1–5). The reaction needs to be carried out at 60 °C for several hours with at least 2 equiv of CuCl_2_. Increasing the amount of CuCl_2_ to 3 equiv did not significantly change the reaction outcome (Table 1, entry 3). Without supplementary oxygen, no product was formed after 3 h at 60 °C. Without supplementary gaseous HCl, only 10% of the desired product was formed after 3 h. Under the optimal conditions listed in Table 1 entry 2, the desired product was converted in 82% and isolated in 75% yield in this 10 mmol trial. It is of interest to note that, even under optimal conditions, such as those listed under entries 2 and 3 in Table 1, no significant amount of any product was formed within the first 30 min or so of the reaction, as indicated by TLC analysis of the reaction mixtures. It seems that there is a kinetic delay of the chlorination reaction.

The best results for the chlorination of 2-methylaniline were obtained with ionic liquids as solvents (Table 1, entries 6–8), which were prepared as reported [12–14]. The conversions to the desired product were detected to be around 90%, and importantly, neither oxygen nor gaseous HCl was needed to achieve a high yield of conversion. The temperature used to carry out the reaction was also lower than that used for the aqueous solvent. The best result was obtained with 1-hexyl-3-methylimidazolium chloride (**3a**) as solvent, in which a 92% conversion with 91% isolated yield was achieved.

In contrast to reactions in aqueous HCl, reactions in ionic liquids did not seem to have a delay in product formation, as product formation could be detected by TLC analysis within minutes of the start of the reaction. In combination with the different requirements for oxygen and gaseous HCl, one may attribute the optimal results in ionic liquids to the efficient dissolution and mixing of reactants. In aqueous solvent, CuCl_2_ is fully solvated by water, but dissolution of organic aniline is sluggish even with the help of HCl. The reaction between the highly solvated Cu(II) and the less optimally solvated aniline is therefore hindered by suboptimal mixing. The produced Cu(I) in the aqueous solvent still hinders the further transformation of the unreacted aniline substrate [5], thus supplementary oxygen is necessary to convert Cu(I) back to Cu(II).

In ionic liquids, Cu(II) and aniline were both dissolved and thus optimally mixed. This allows the reaction to proceed at maximum speed without delay. In addition, Cu(I) produced during the reaction did not hinder the reaction with the unreacted aniline, thus, no supplement of oxygen was necessary during the reaction. In fact, in our experience, with 3 or 4 equiv of CuCl_2_, one can repeatedly perform the chlorination reaction two or three times, respectively, with no loss of efficiency before regeneration of Cu(II). In ionic liquids, chloride as a counter ion of the solvent is in large excess, and Cu(II) regeneration can be performed offline by passing either oxygen or simply air through the ionic liquid that contains the Cu(I)/Cu(II) mixture, to restore full reaction power. This important feature makes the chlorination in ionic liquids much more attractive than in aqueous HCl in terms of operational safety and environmental impact.

Further expansion of the chlorination reaction to other aniline analogous was conducted in 1-hexyl-3-methylimidazolium chloride (**3a**), as summarized in Table 2. All reactions were attempted on larger scales (100 mmol, about 10 g) than the initial testing. As shown in Table 2, for all 2- or 3-mono-substituted anilines excellent results were obtained in terms of isolated yields of the desired 4-Cl products, which ranged from 85–96% (Table 2, entries 1–9, see Supporting Information File 1). The substitutions ranged from electron-donating substituents, such as the methoxy group, to electron-withdrawing ones, such as the nitro group. It is not surprising that a short reaction time (3–4 h) was sufficient for the anilines with electron-donating substituents, but a longer time (up to 16 h) was necessary for those with electron-withdrawing substituents. When 4-substituted anilines were used (entries 10 and 11), no chlorination products were detected or isolated, indicating that the most stable resonance of the aniline cation generated by Cu(II) oxidation had the positive charge at the C4-position. 2-Aminopyridine did not give any product under similar reaction conditions.

**Table 2:** *para*-Chlorination of aniline analogues (100 mmol scale) using CuCl_2_ in 1-hexyl-3-methylimidazolium chloride (**3a**).^a^

entry	substrate	time	yield of 4-Cl product^b^	isomer detected in crude mixture^c^

1	2-methylaniline (**2a**)	4 h	**4a**, 91%	2.5%
2	2-methoxyaniline (**2b**)	3 h	**4b**, 93%	2.7%
3	2-fluoroaniline (**2c**)	4 h	**4c**, 88%	2.0%
4	2-trifluoromethylaniline (**2d**)	6 h	**4d**, 90%	2.2%
5	2-nitroaniline (**2e**)	16 h	**4e**, 85%	1.8%
6	3-methylaniline (**2f**)	4 h	**4f**, 95%	1.7%
7	3-methoxyaniline (**2g**)	3 h	**4g**, 96%	2.2%
8	3-fluoroaniline (**2h**)	6 h	**4h**, 92%	1.9%
9	3-trifluoromethylaniline (**2i**)	8 h	**4i**, 94%	1.6%
10	4-nitroaniline (**2j**)	4 h	**4j**, —	—
11	4-methoxyaniline (**2k**)	4 h	**4k**, —	—
12	2-aminopyridine (**2l**)	4 h	**4l**, —	—

^a^All reactions were carried out with 3 equiv of CuCl_2_ at 40 °C. ^b^Isolated yields. ^c^Based on GC–MS analysis of crude reaction mixtures.

With the success of chlorination in ionic liquids, we also attempted to extend the reaction to other halides. While attempts to use fluoride and iodide failed, bromination was successfully achieved in high yields and regioselectivity, in an ionic liquid with bromide as a counter ion (Table 3). In general, the reaction time for bromination was shorter than that for the corresponding chlorination reaction, and was achieved at lower temperature (room temperature for bromination). In all cases, as shown in Table 3, high regioselectivity of para-bromination was again achieved, similar to the chlorination results.

**Table 3:** Bromination of aniline analogues (100 mmol scale) using CuBr_2_ in 1-hexyl-3-methylimidazolium bromide.^a^

entry	substrate	time	yield of 4-Br product^b^	isomer detected in crude mixture^c^

1	2-methylaniline (**2a**)	1 h	**5a**, 95%	—
2	2-methoxyaniline (**2b**)	1 h	**5b**, 95%	—
3	2-fluoroaniline (**2c**)	0.5 h	**5c**, 91%	1.2%
4	2-trifluoromethylaniline (**2d**)	1 h	**5d**, 92%	2.8%
5	2-nitroaniline (**2e**)	3 h	**5e**, 88%	—
6	3-methylaniline (**2f**)	1 h	**5f**, 95%	2.5%
7	3-methoxyaniline (**2g**)	1 h	**5g**, 95%	2.2%
8	3-fluoroaniline (**2h**)	10 min	**5h**, 90%	—
9	3-trifluoromethylaniline (**2i**)	1 h	**5i**, 93%	2.4%

^a^All reactions were carried out with 3 equiv of CuBr_2_ at rt. ^b^Isolated yields. ^c^Based on GC–MS analysis of crude reaction mixtures.

## Conclusion

In summary, we showed that using an ionic liquid as a solvent, the direct chlorination or bromination of unprotected aniline derivatives using CuCl_2_ or CuBr_2_ can be achieved in high yield and high regioselectivity (para-substitution in most cases) under mild conditions without the need for supplementary oxygen or HCl gas. Thus, our new protocol offers a safer operational choice and reduced environmental impact when compared to direct chlorination in aqueous HCl, or halogenation of protected/masked anilines, which requires additional deprotection processes.

## Supporting Information

File 1 Experimental section and characterization data.

File 2 NMR spectra of all compounds.
