# Supplementary material for: Regioselective chlorination and bromination of unprotected anilines under mild conditions using copper halides in ionic liquids
Source: Beilstein J Org Chem. 2012 May 16;8:744–8. doi: 10.3762/bjoc.8.84 (PMC3388862; doi:10.3762/bjoc.8.84)
Supplement: File 1 — Experimental section and characterization data. [file Beilstein_J_Org_Chem-08-744-s001.pdf]

Supporting Information

for

Regioselective chlorination and bromination of unprotected anilines under mild conditions using copper halides in ionic liquids

Han Wang[1,2], Kun Wen[2], Nurbiya Nurahmat[2], Yan Shao[2], He Zhang[2], Chao Wei[2], Ya Li[2], Yongjia Shen[*1] and Zhihua Sun[*2]

Address: [1]School of Chemistry and Molecular Engineering, East China University of Science and Technology, Shanghai, 200237, China and [2]College of Chemistry and Chemical Engineering, Shanghai University of Engineering Science, Shanghai, 201620, China

Email: Yongjia Shen - yjshen@ecust.edu.cn; Zhihua Sun* - zhihuasun@sues.edu.cn

* Corresponding author

Experimental section and characterization data

Experimental section

All reactions were carried out using commercial solvents without further purification, unless otherwise noted. Ionic liquids were prepared as reported. GC–MS analysis was performed with a Finnigan Trace GC-DSQ system, using a 30 m length DB-5 MS column at 50–250 °C. HPLC was performed with an Agilent 1200 system using a C18 reverse-phase column (5 m, 4.6 by 250 mm) and monitored at 230 nm with aqueous TFA and CH_3CN gradients. NMR was recorded on a Bruker instrument at 400 MHz for ^{1}H, 100 MHz for ^{13}C, and 376 MHz for ^{19}F.

Chlorination in ionic liquid: To a 500 mL flask containing 150 mL 1-hexyl-3-methylimidazolium chloride (**3a**), 40 g (0.3 mol) $CuCl_2$ was added. The mixture was sonicated to a homogenous solution and heated to 40 °C. Then 10.7 g (0.1 mol) 2-methylaniline (**2a**) was added. The reaction was allowed to proceed for 4 h. The reaction mixture was extracted with 50 mL ethyl acetate twice. The combined organic phase was washed and dried. After reduction of the volume of ethyl acetate and recrystallization with the addition of petroleum ether, the desired product **4a** was obtained as a white crystalline solid. Yield: 12.8 g (91%). Purity (HPLC at 230 nm): 97%.

4-Chloro-2-methylaniline (**4a**)**.** Reaction was carried out for 4 h. Yield: 12.8 g (91%). Purity (HPLC at 230 nm): 97%; mp 28–29 °C; ^{1}H NMR ($CDCl_3$, δ, ppm) 7.07 (s, 1H), 7.04 (d, J = 8.4 Hz, 1H), 6.59 (d, J = 8.4 Hz, 1H), 3.63 (br s, 2H), 2.15 (s, 3H); ^{13}C NMR ($CDCl_3$, δ, ppm) 143.4, 130.0, 126.7, 124.1, 122.8, 116.0, 17.2; EIMS *m*/*z* (% relative intensity): 140.87 (100%), 142.88 (32%).

4-Chloro-2-methoxyaniline (**4b**)**.** Reaction was carried out for 3 h. Yield: 14.6 g (93%). Purity: 99% (HPLC at 230 nm); mp 52–53 °C; ^{1}H NMR ($CDCl_3$, δ, ppm) 6.80 (s, 1H), 6.79 (d, J = 8.8 Hz, 1H), 6.61 (d, J = 8.8 Hz, 1H), 3.86 (br s, 2H), 3.81 (s, 3H); ^{13}C NMR ($CDCl_3$, δ, ppm) 147.7, 135.0, 122.6, 120.7, 115.3, 111.0, 55.6; EIMS *m*/*z* (% relative intensity): 156.87 (100%), 158.90 (32%).

4-Chloro-2-fluoroaniline (**4c**)**.** Reaction was carried out for 4 h. Yield: 12.8 g (88%) as an oil. Purity: 96% (HPLC at 230 nm); ^{1}H NMR ($CDCl_3$, δ, ppm) 7.03 (d, J = 10.8 Hz, 1H), 6.94 (d, J = 8.0 Hz, 1H), 6.72 (t, J = 9.2 Hz, J = 8.8 Hz, 1H), 3.74 (br s, 2H); ^{13}C NMR ($CDCl_3$, δ, ppm) 151.2, 133.3, 124.5, 122.5, 117.3, 116.9; ^{19}F NMR ($CDCl_3$, δ, ppm) −132.4; MS (EI, m/z): 144.90 (100%), 146.91 (32%).

4-Chloro-2-trifluoromethylaniline (4d). Reaction was carried out for 6 h. Yield: 17.6 g (90%) as an oil. Purity: 98.5% (HPLC at 230 nm); ^{1}H NMR ($CDCl_3$, δ, ppm) 7.42 (s, 1H), 7.24 (d, *J* = 8.4 Hz, 1H), 6.66 (d, *J* = 8.8 Hz, 1H), 4.20 (br s, 2H); ^{13}C NMR ($CDCl_3$, δ, ppm) 145.7, 133.3, 126.1, 125.9, 124.7, 119.1, 118.8; ^{19}F NMR ($CDCl_3$, δ, ppm) −62.1. EIMS *m*/*z* (% relative intensity): 194.87 (100%), 196.88 (32%).

4-Chloro-2-nitroaniline (4e). Reaction was carried out for 16 h. Yield: 14.6 g (85%). Purity: 94.5% (HPLC at 230 nm); mp 96–98 °C; ^{1}H NMR ($CDCl_3$, δ, ppm) 8.15 (s, 1H), 7.34 (d, *J* = 8.8 Hz, 1H), 6.80 (d, *J* = 8.8 Hz, 1H), 6.12 (br s, 2H); ^{13}C NMR ($CDCl_3$, δ, ppm) 143.2, 135.9, 132.2, 125.4, 121.6, 120.0; EIMS *m*/*z* (% relative intensity): 171.83 (100%), 173.84 (32%).

4-Chloro-3-methylaniline (4f). Reaction was carried for 4 h. Yield: 13.4 g (95%). Purity: 99% (HPLC at 230 nm); mp 92–94 °C; ^{1}H NMR ($CDCl_3$, δ, ppm) 7.11 (d, *J* = 8.4 Hz, 1H), 6.58 (s, 1H); 6.48 (d, *J* = 8.0 Hz, 1H), 6.61 (d, *J* = 8.8 Hz, 1H), 3.61 (br s, 2H), 2.30 (s, 3H); ^{13}C NMR ($CDCl_3$, δ, ppm) 145.0, 136.6, 129.5, 123.5, 117.5, 113.9, 20.1; EIMS *m*/*z* (% relative intensity): 156.87 (100%), 158.90 (33%).

4-Chloro-3-methoxyaniline (4g). Reaction was carried out for 3 h. Yield: 15.1 (96%). Purity: 99% (HPLC at 230 nm); mp 78–80 °C; ^{1}H NMR ($CDCl_3$, δ, ppm) 7.11 (d, *J* = 8.4 Hz, 1H), 6.29 (s, 1H), 6.24 (d, *J* = 8.4 Hz, 1H), 3.86 (s, 3H), 3.72 (br s, 2H); ^{13}C NMR ($CDCl_3$, δ, ppm) 155.6, 146.5, 130.4, 111.5, 107.8, 99.8, 55.9; EIMS *m*/*z* (% relative intensity): 156.81 (100%), 158.87 (34%).

4-Chloro-3-fluoroaniline (4h). Reaction was carried out for 6 h. Yield: 13.1 g (92%). Purity: 99% (HPLC at 230 nm); mp 62–63 °C; ^{1}H NMR ($CDCl_3$, δ, ppm) 7.13 (t, *J* = 8.4 Hz, *J* = 8.4 Hz, 1H), 6.47 (d, *J* = 11.2 Hz, 1H), 6.41 (d, *J* = 8.4 Hz, 1H), 3.79 (br s, 2H); ^{13}C NMR ($CDCl_3$, δ, ppm) 158.6, 146.8, 130.8, 111.4, 109.4, 103.2; ^{19}F NMR ($CDCl_3$, δ, ppm) −115.3; EIMS *m*/*z* (% relative intensity): 144.77 (100%), 146.85 (38%).

4-Chloro-3-trifluoromethylaniline (4i). Reaction was carried out for 8 h. Yield: 18.3 g (94%). Purity: 99% (HPLC at 230 nm); mp 36–37 °C; ^{1}H NMR ($CDCl_3$, δ, ppm) 7.23 (d, *J* = 6.4 Hz, 1H), 6.96 (s, 1H), 6.73 (d, *J* = 6.4 Hz, 1H), 3.88 (br s, 2H); ^{13}C NMR ($CDCl_3$, δ, ppm) 145.3, 132.1, 128.7, 123.0, 120.1, 118.7, 113.6; ^{19}F NMR ($CDCl_3$, δ, ppm) −62.7. EIMS *m*/*z* (% relative intensity): 194.82 (100%), 196.85 (32%).

Bromination in ionic liquid: To a 500 mL flask containing 150 mL 1-hexyl-3-methylimidazolium bromide, was added 33.4 g (0.15 mol) $CuBr_2$. The mixture was sonicated to a homogenous solution and heated to 40 °C. Then 5.4 g (0.05 mol) 2-methylaniline (**2a**) was added. The reaction was allowed to proceed for 1 h. The reaction mixture was extracted with 50 mL ethyl acetate twice. The combined organic phase was washed and dried. After reducing ethyl acetate volume and recrystallization with the addition of petroleum ether, the desired product **5a** was obtained as a white crystalline solid. Yield: 8.8 g (95%). Purity (HPLC at 230 nm): 97%.

4-Bromo-2-methylaniline (5a). Reaction was carried out for 1 h. Yield: 95%. Purity: 97% (HPLC at 230 nm); mp 56–57 °C; ^{1}H NMR ($CDCl_3$, δ, ppm) 7.19 (d, *J* = 2 Hz, 1H), 7.14 (dd, *J* = 2 Hz, *J* = 8.4 Hz, 1H), 6.56 (d, *J* = 8.4 Hz, 1H), 3.61 (br s, 2H), 2.16 (s, 3H); ^{13}C NMR ($CDCl_3$, δ, ppm) 143.7, 132.8, 129.6, 124.4, 116.4, 110.1, 17.2; EIMS *m*/*z* (% relative intensity): 184.97 (100%), 186.97 (97%).

4-Bromo-2-methoxyaniline (5b). Reaction was carried out for 1 h. Yield: 19.2 g (95%). Purity: 99% (HPLC at 230 nm); mp 61–62 °C; ^{1}H NMR ($CDCl_3$, δ, ppm) 6.93 (d, *J* = 2.4 Hz, 1H), 6.91 (s, 1H), 6.60 (dd, *J* = 0.8 Hz, *J* = 7.6 Hz, 1H), 3.86 (s, 3H), 3.80 (br s, 2H); ^{13}C NMR ($CDCl_3$, δ, ppm) 147.9, 135.4, 123.7, 115.7, 113.8, 109.6, 55.7; EIMS *m*/*z* (% relative intensity): 200.98 (100%), 202.98 (97%).

4-Bromo-2-fluoroaniline (5c). Reaction was carried out for 0.5 h. Yield: 17.3 g (91%). Purity: 96% (HPLC at 230 nm); mp 40–41°C; ^{1}H NMR ($CDCl_3$, δ, ppm) 7.16 (dd, *J* = 2 Hz, *J* = 10.4 Hz, 1H), 7.06 (m, 1H), 6.66 (td, *J* = 0.8 Hz, *J* = 9.6 Hz, 1H), 3.69 (br s, 2H); ^{13}C NMR ($CDCl_3$, δ, ppm) 151.4, 133.8, 127.4, 118.7, 117.8, 108.9; ^{19}F NMR ($CDCl_3$, δ, ppm) −132.2; EIMS *m*/*z* (% relative intensity): 188.96 (100%), 190.96 (98%).

4-Bromo-2-trifluoromethylaniline (5d). Reaction was carried out for 1 h. Yield: 22.1 g oil (92%). Purity: 98% (HPLC at 230 nm); ^{1}H NMR ($CDCl_3$, δ, ppm) 7.56 (d, *J* = 2 Hz, 1H), 7.39 (dd, *J* = 2 Hz, *J* = 8.8 Hz, 1H), 6.64 (d, *J* = 8.8 Hz, 1H), 4.17 (br s, 2H); ^{13}C NMR ($CDCl_3$, δ, ppm) 143.5, 135.6, 129.2, 124.1, 118.8, 115.3; ^{19}F NMR($CDCl_3$, δ, ppm) −63.2. EIMS *m*/*z* (% relative intensity): 238.96 (100%), 240.96 (97%).

4-Bromo-2-nitroaniline (**5e**)**.** Reaction was carried out for 3 h. Yield: 19.1 g (88%). Purity: 96% (HPLC at 230 nm); mp 112–114 °C; ^{1}H NMR ($CDCl_3$, δ, ppm) 8.29 (d, *J* = 2.4 Hz, 1H), 7.45 (dd, *J* = 2.4 Hz, *J* = 8.8 Hz, 1H), 6.75 (d, *J* = 8.8 Hz, 1H), 6.11 (br s, 2H); ^{13}C NMR (CDCl3, δ, ppm) 143.6, 138.4, 132.6, 128.3, 120.3, 107.8; EIMS *m*/*z* (% relative intensity): 215.95 (100%), 217.95 (97%).

4-Bromo-3-methylaniline (**5f**)**.** Reaction was carried out for 1 h. Yield: 17.7 g (95%). Purity: 99% (HPLC at 230 nm); mp 78–80 °C; ^{1}H NMR ($CDCl_3$, δ, ppm) 7.28 (d, *J* = 8.4 Hz, 1H), 6.59 (d, *J* = 2.4 Hz, 1H), 6.42 (dd, *J* = 2.4 Hz, *J* = 8.4 Hz, 1H), 3.60 (br s, 2H), 2.32 (s, 3H); ^{13}C NMR ($CDCl_3$, δ, ppm) 145.6, 138.4, 132.7, 117.5, 114.3, 113.0, 22.8; EIMS *m*/*z* (% relative intensity): 184.97 (100%), 186.97 (97%).

4-Bromo-3-methoxyaniline (**5g**)**.** Reaction was carried out for 1 h. Yield: 19.2 g (95%). Purity: 99% (HPLC at 230 nm); mp 94–95 °C; ^{1}H NMR ($CDCl_3$, δ, ppm) 7.30 (d, *J* = 8.8 Hz, 1H), 6.34 (d, J = 2.8 Hz, 1H), 6.25 (dd, *J* = 2.8 Hz, *J* = 8.8 Hz, 1H), 4.10 (br s, 2H), 3.76 (s, 3H); ^{13}C NMR ($CDCl_3$, δ, ppm) 160.1, 144.9, 132.9, 105.6, 101.4, 100.5, 55.4; EIMS *m*/*z* (% relative intensity): 200.98 (100%), 202.98 (97%).

4-Bromo-3-fluoroaniline (**5h**)**.** Reaction was carried out for 10 min. Yield: 17.1 g (90%). Purity: 99% (HPLC at 230 nm); mp 188–190 °C; ^{1}H NMR ($CDCl_3$, δ, ppm) 7.26 (t, *J* = 8.4 Hz, *J* = 8.0 Hz, 1H), 6.47 (dd, *J* = 2.8 Hz *J* = 10.4 Hz, 1H), 6.37 (dd, *J* = 2.0 Hz, *J* = 8.4 Hz, 1H), 3.80 (br s, 2H); ^{13}C NMR ($CDCl_3$, δ, ppm) 159.6, 147.6, 133.5, 112.0, 103.2, 96.2; ^{19}F NMR ($CDCl_3$, δ, ppm) −107.4; EIMS *m*/*z* (% relative intensity): 188.96 (100%), 190.96 (98%).

4-Bromo-3-trifluoromethylaniline (**5i**)**.** Reaction was carried out for 1 h. Yield: 22.6 g (93%). Purity: 99% (HPLC at 230 nm); mp 52–54 °C; ^{1}H NMR ($CDCl_3$, δ, ppm) 7.52 (d, *J* = 8.4 Hz, 1H), 6.99 (d, *J* = 2 Hz, 1H), 6.88 (dd, *J* = 2 Hz, *J* = 8.4 Hz, 1H), 4.31 (br s, 2H); ^{13}C NMR ($CDCl_3$, δ, ppm) 144.5, 133.1, 130.9, 123.9, 115.6, 112.4, 111.9; ^{19}F NMR ($CDCl_3$, δ, ppm) −62.9; EIMS *m*/*z* (% relative intensity): 238.96 (100%), 240.96 (97%).

Attempted fluorination in ionic liquid: To a 500 mL flask containing 150 mL 1-hexyl-3-methylimidazolium chloride, was added 30.3 g (0.3 mol) CuF_2. Then 10.7 g (0.1 mol) 2-methylaniline (**2a**) was added. The mixture was sonicated to a homogenous solution and heated to 40–200 °C. The

reaction was allowed to proceed for 2–4 h. The reaction mixture was extracted with 50 mL ethyl acetate twice. There was no product detected by GC–MS analysis.

Attempted iodination in ionic liquid： To a 500 mL flask containing 150 mL 1-hexyl-3-methylimidazolium chloride, was added 16.4 g (0.15 mol) CuI. The mixture was sonicated to a homogenous solution and heated to 40 °C. Then 5.4 g (0.05 mol) 2-methylaniline (**2a**) was added. The reaction was allowed to proceed for 2 h. The reaction mixture was extracted with 50 mL ethyl acetate twice. There was no product detected by GC–MS analysis.
