# Supplementary material for: Regioselective chlorination and bromination of unprotected anilines under mild conditions using copper halides in ionic liquids
Source: Beilstein J Org Chem. 2012 May 16;8:744–8. doi: 10.3762/bjoc.8.84 (PMC3388862; doi:10.3762/bjoc.8.84)

Supporting Information

for

Regioselective chlorination and bromination of unprotected anilines under mild conditions using copper halides in ionic liquids

Han Wang[1,2], Kun Wen[2], Nurbiya Nurahmat[2], Yan Shao[2], He Zhang[2], Chao Wei[2], Ya Li[2], Yongjia Shen[*1] and Zhihua Sun[*2]

Address: [1]School of Chemistry and Molecular Engineering, East China University of Science and Technology, Shanghai, 200237, China and [2]College of Chemistry and Chemical Engineering, Shanghai University of Engineering Science, Shanghai, 201620, China

Email: Yongjia Shen - yjshen@ecust.edu.cn; Zhihua Sun* - zhihuasun@sues.edu.cn

* Corresponding author

NMR spectra of all compounds

4-CHLORO-2-METHYLANILINE (**4a**): ^{1}H NMR

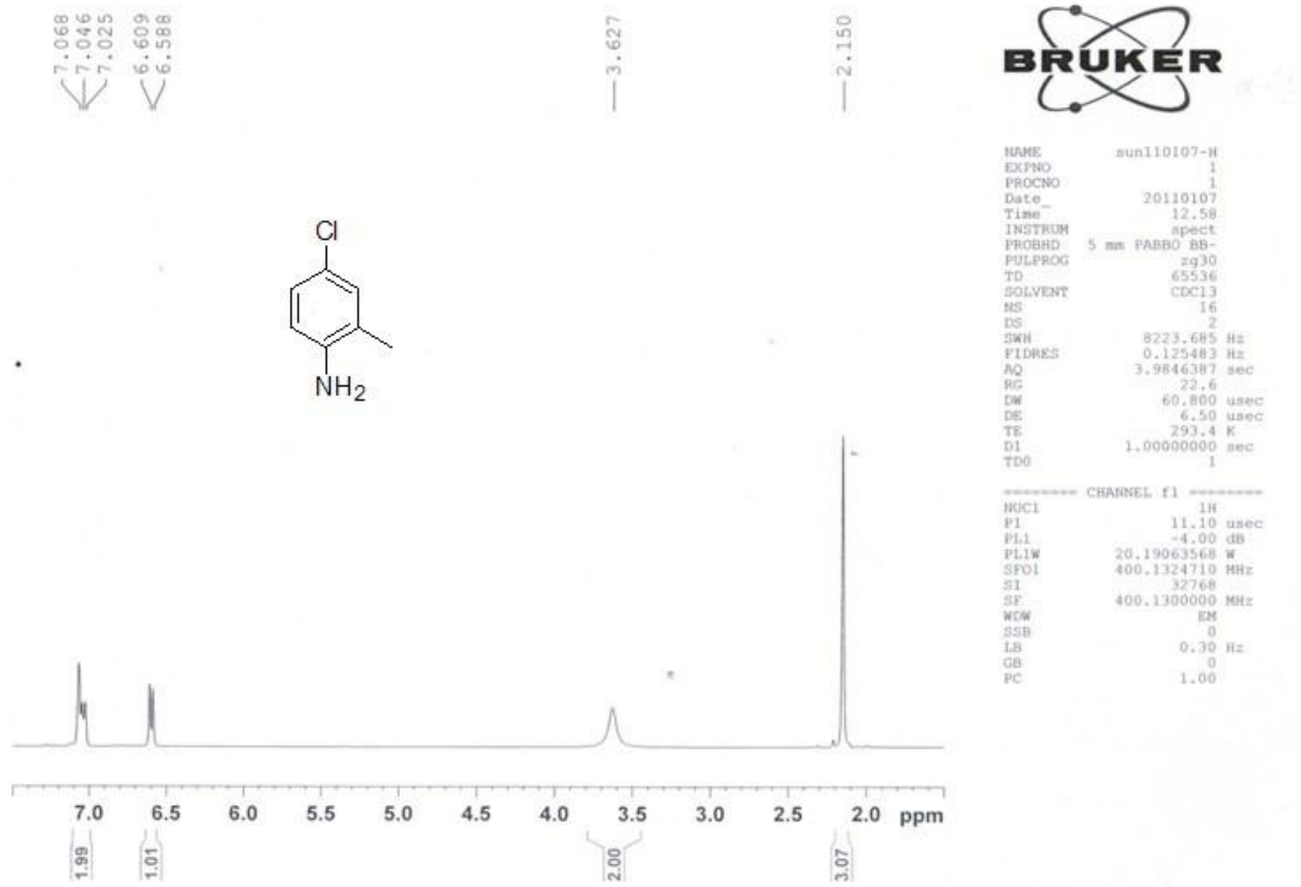

4-CHLORO-2-METHYLANILINE (**4a**): ^{13}C NMR

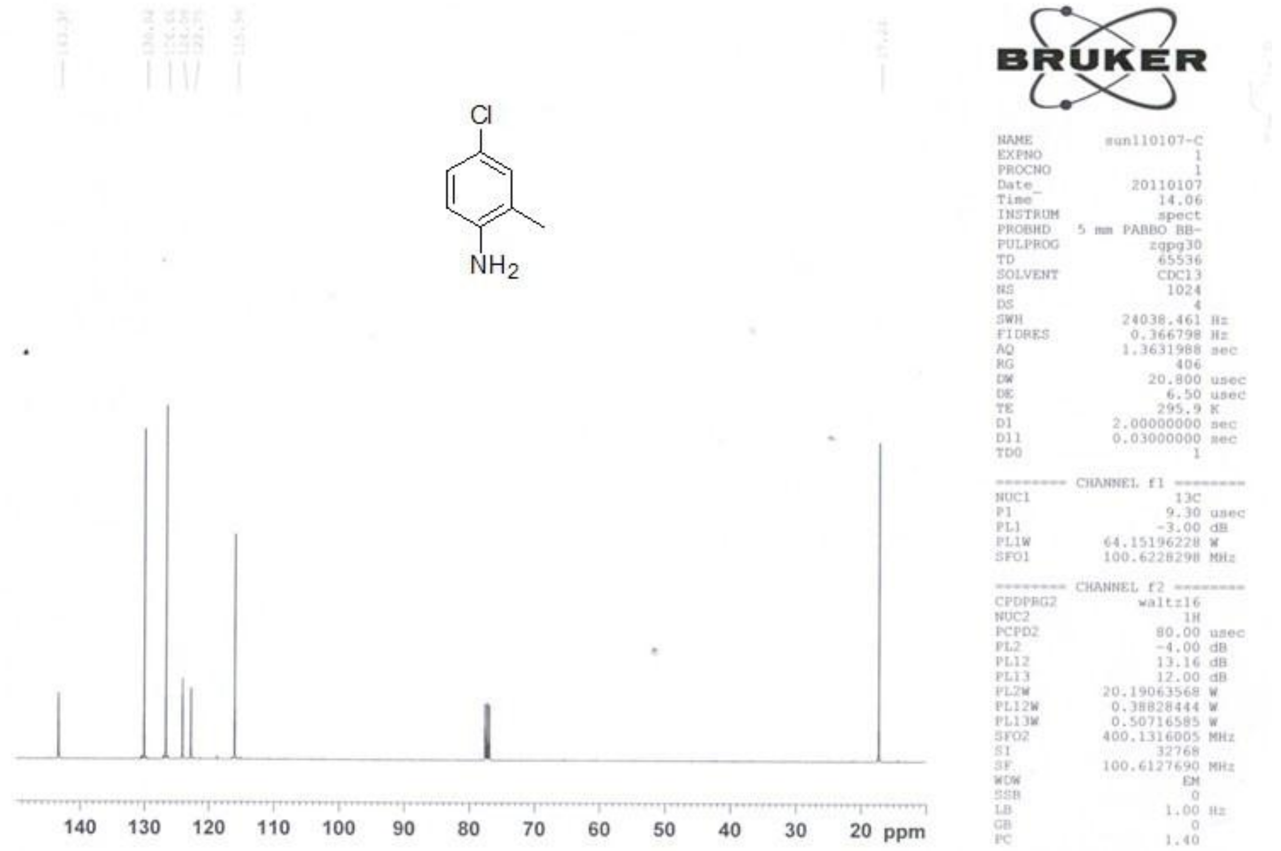

4-CHLORO-2-METHOXYANILINE (**4b**): ^{1}H NMR

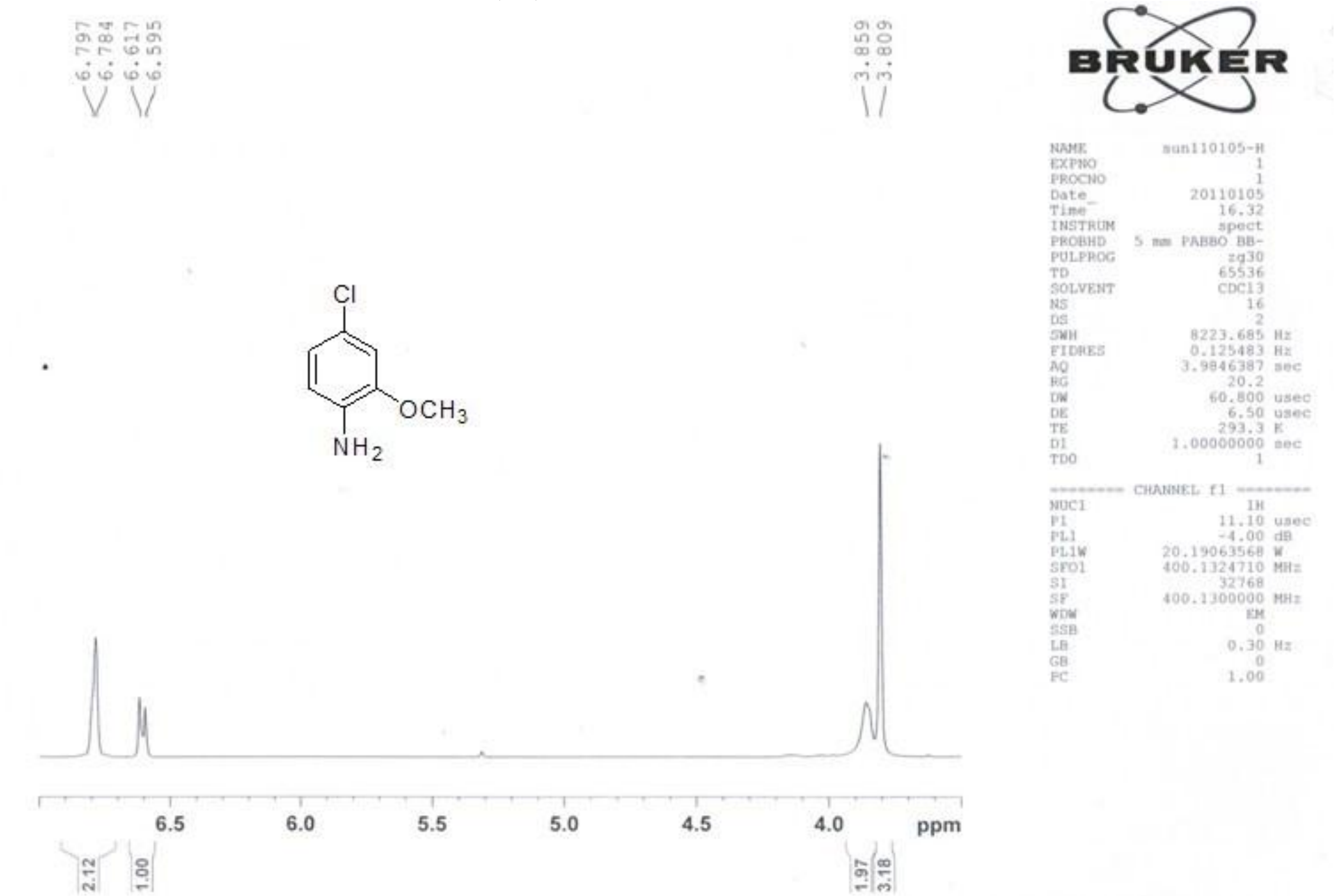

4-CHLORO-2-METHOXYANILINE (**4b**): ^{13}C NMR

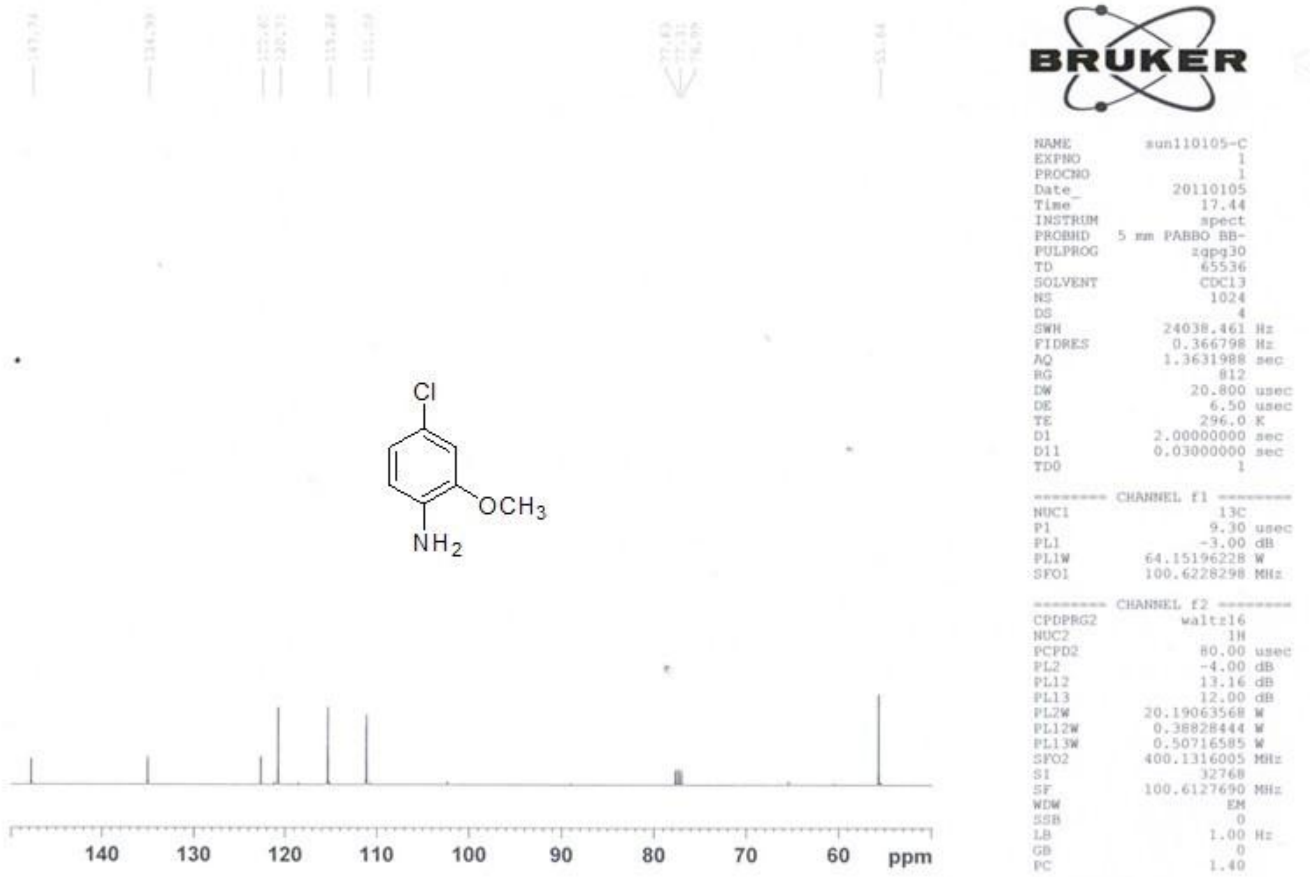

4-CHLORO-2-FLUOROANILINE (**4c**):^{1}H NMR

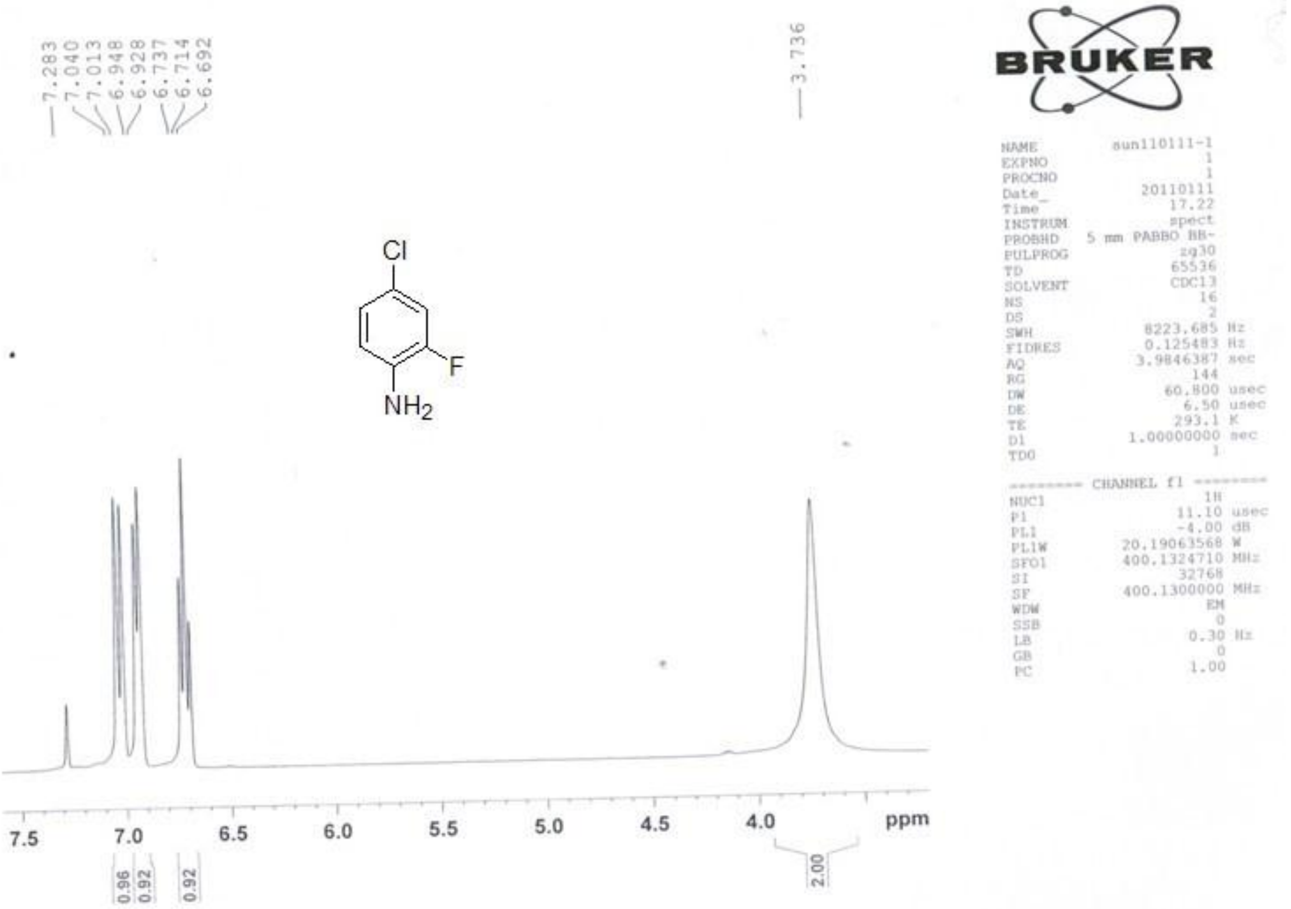

4-CHLORO-2-FLUOROANILINE (**4c**): ^{13}C NMR

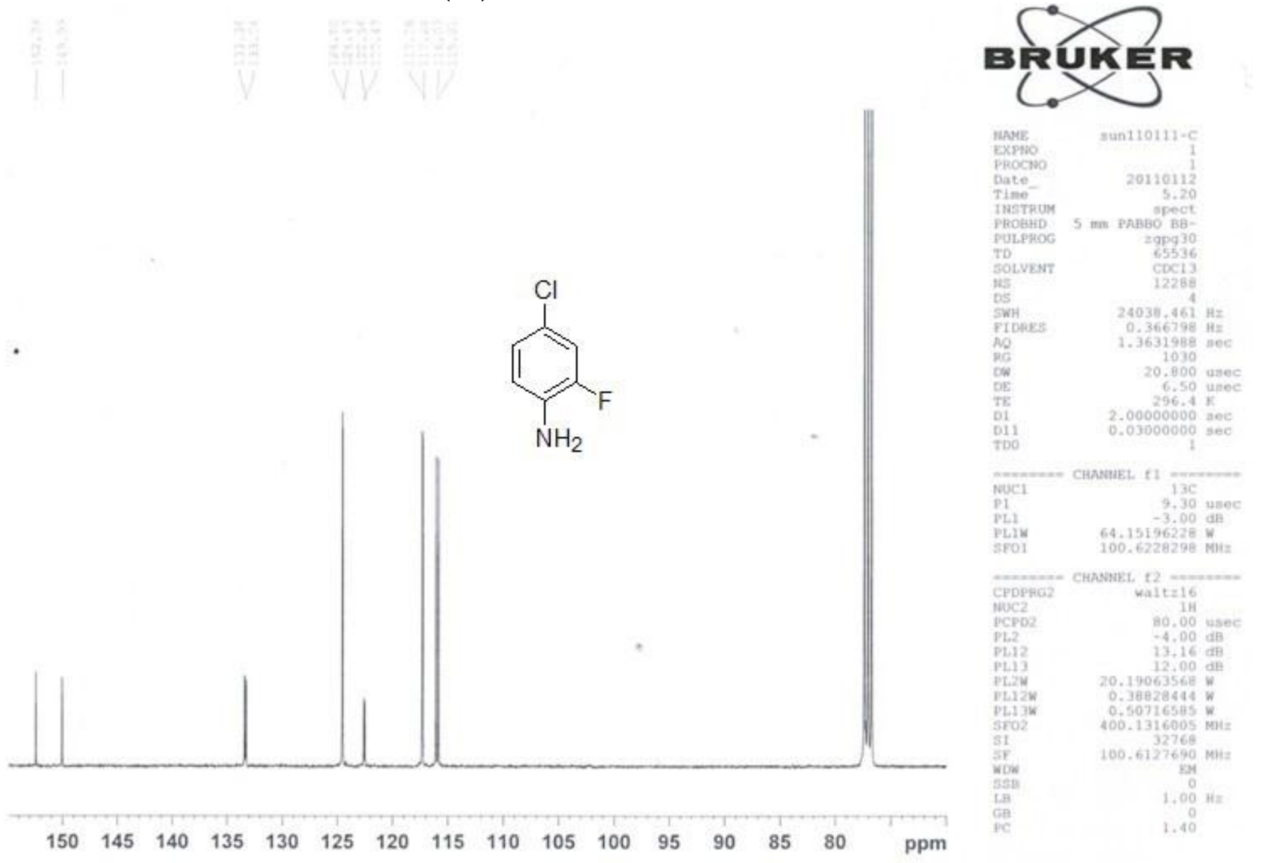

4-CHLORO-2-FLUOROANILINE (**4c**): ^{19}F NMR

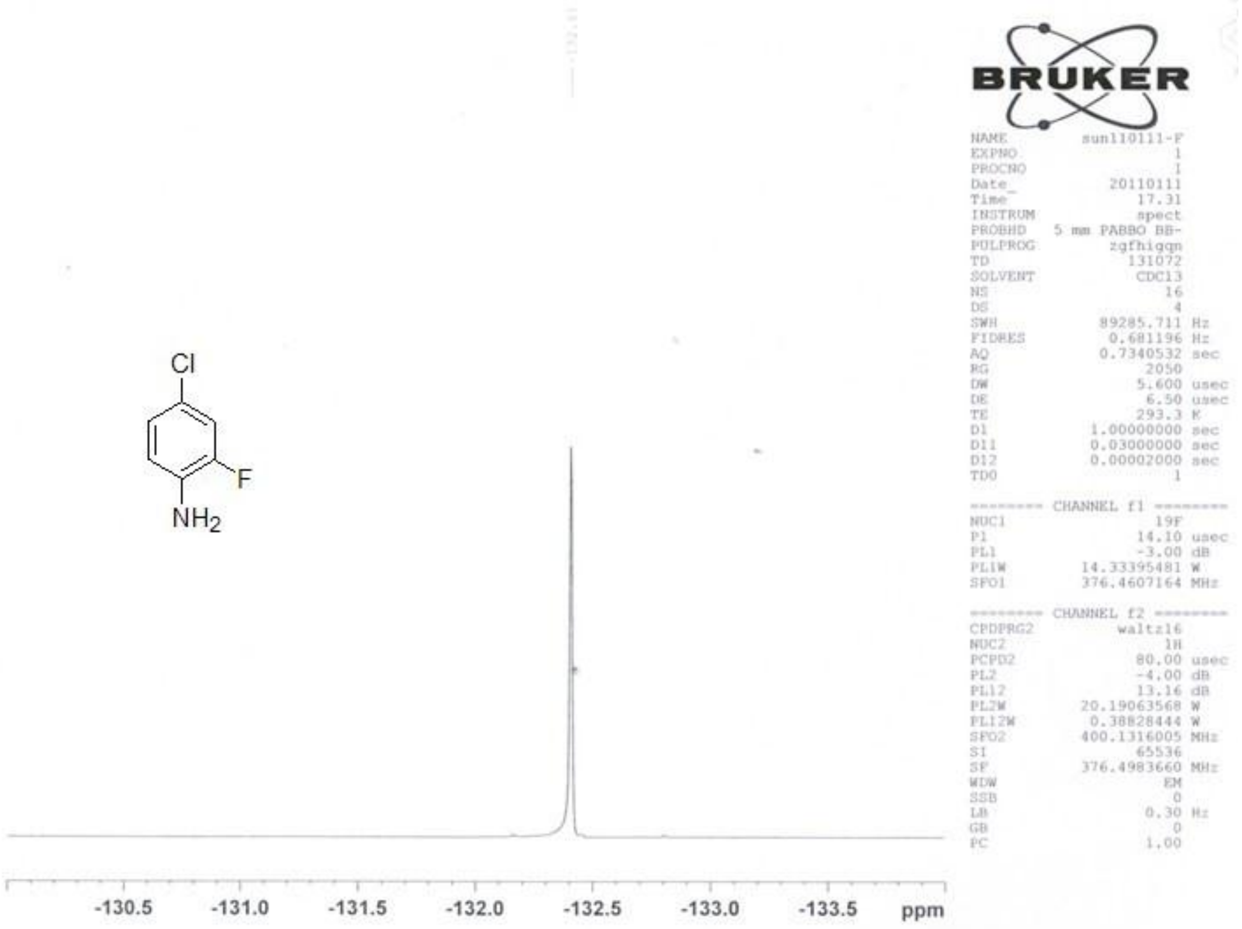

4-CHLORO-2-TRIFLUOROMETHYLANILINE (**4d**): ^{1}H NMR

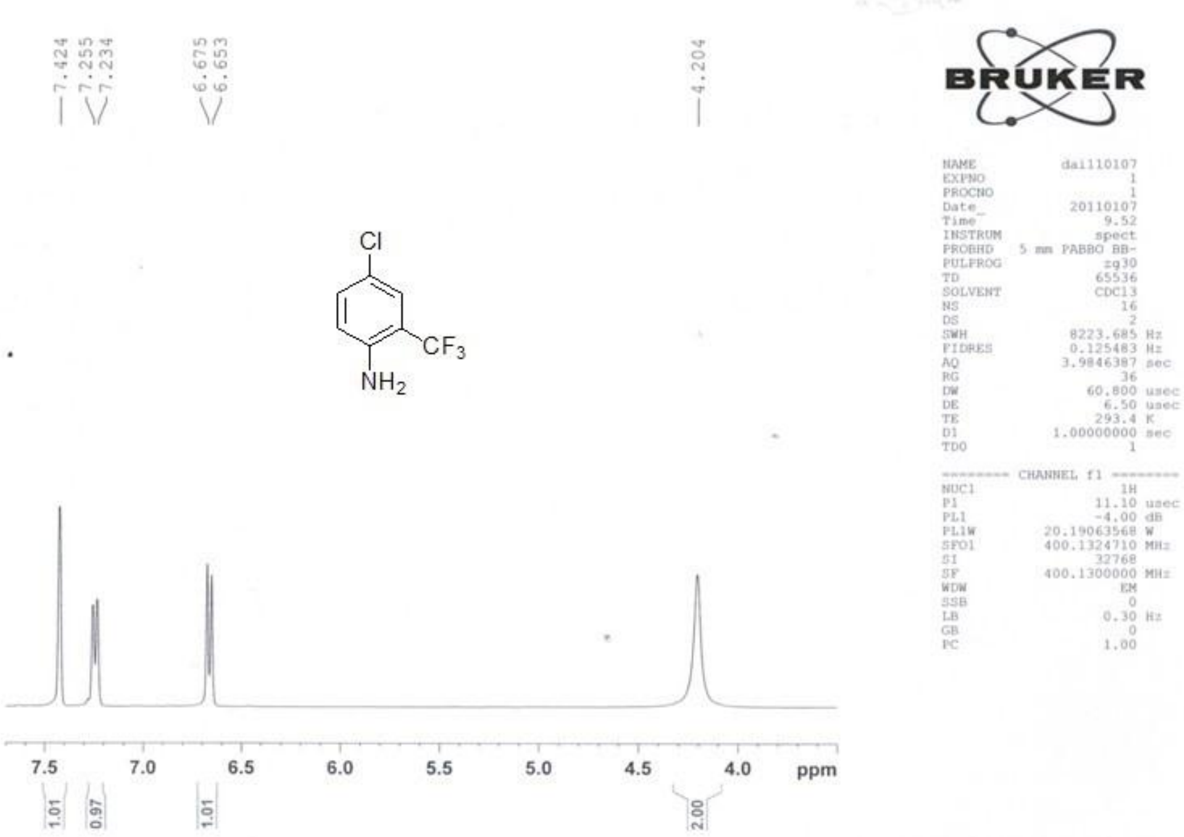

4-CHLORO-2-TRIFLUOROMETHYLANILINE (**4d**): ^{13}C NMR

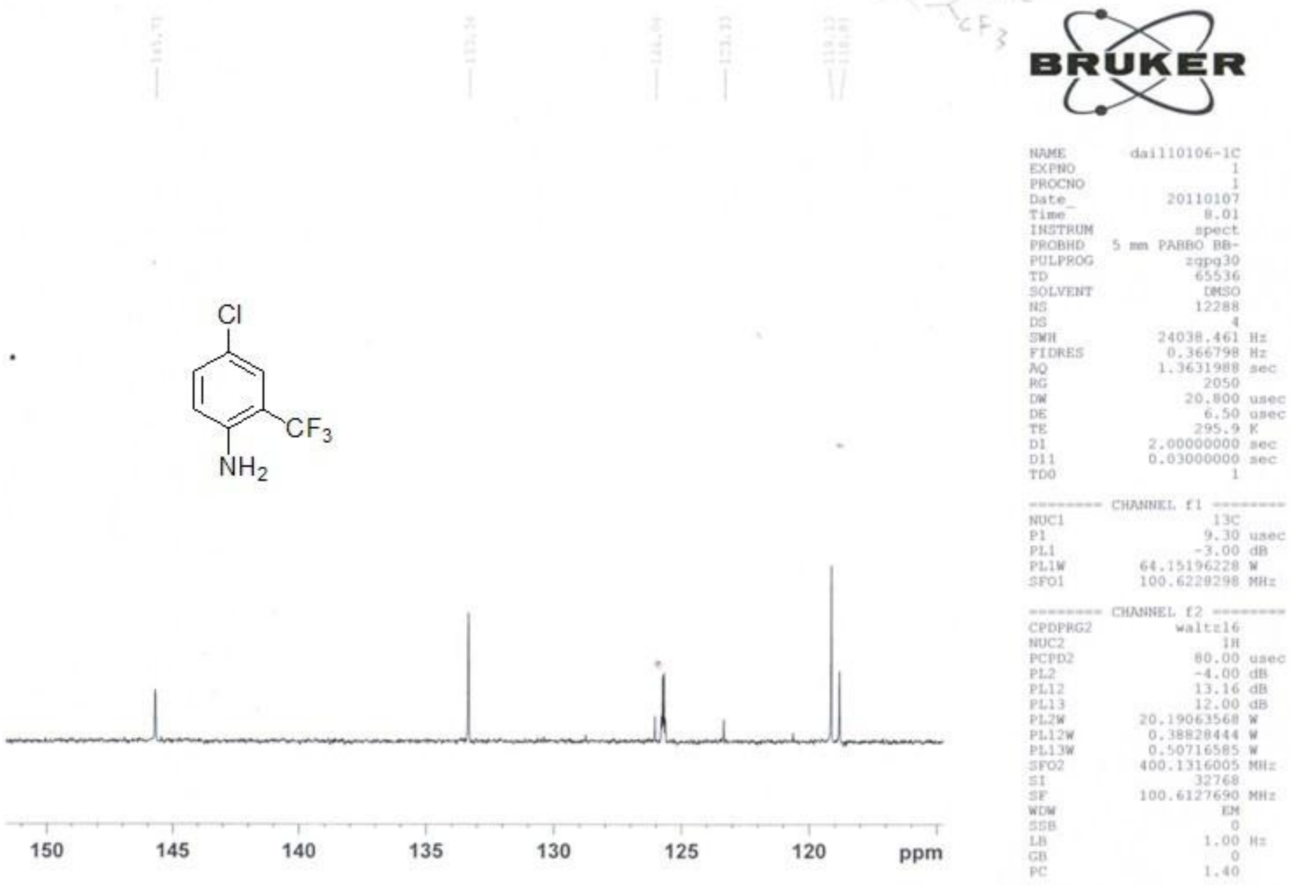

4-CHLORO-2-TRIFLUOROMETHYLANILINE (**4d**): ^{19}F NMR

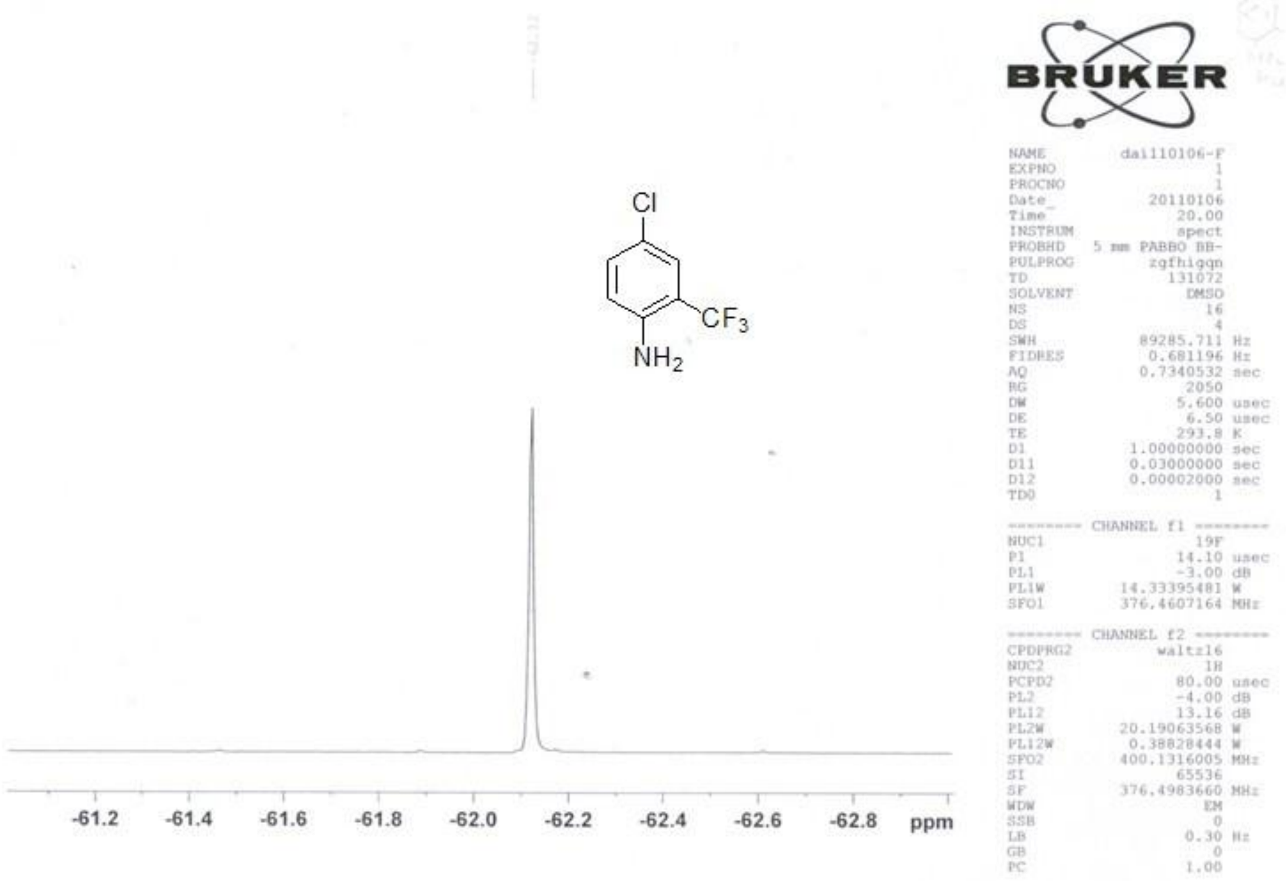

4-CHLORO-2-NITROANILINE (**4e**): ^{1}H NMR

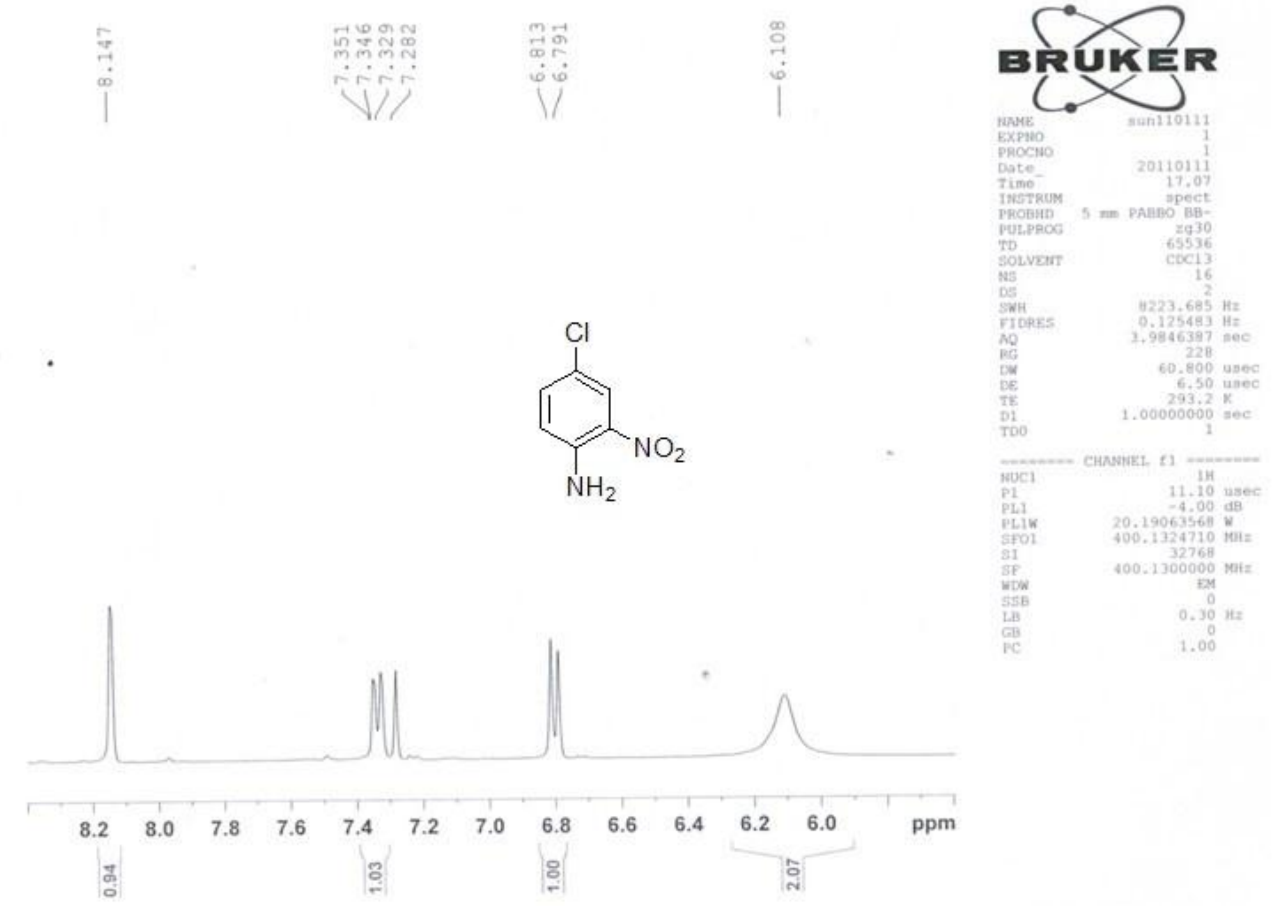

4-CHLORO-2-NITROANILINE (**4e**): ^{13}C NMR

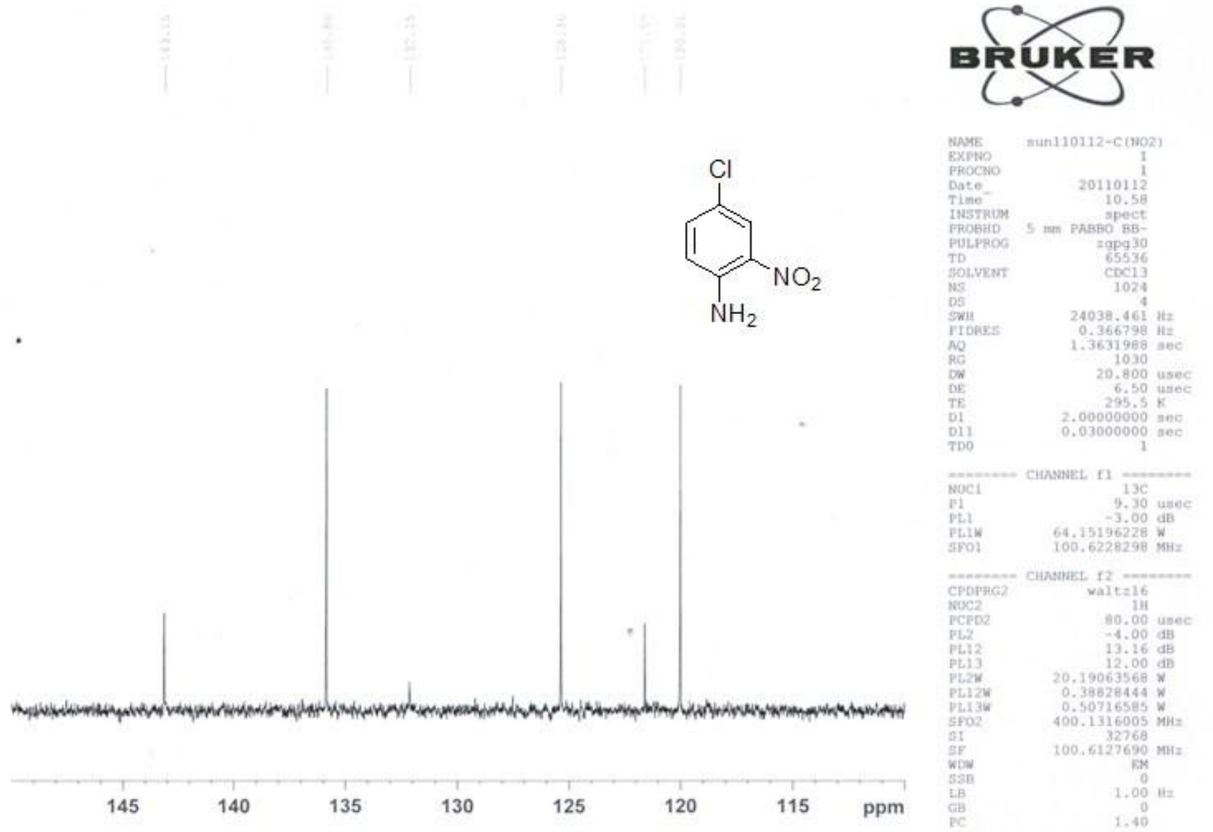

4-CHLORO-3-METHYLANILINE (**4f**): ^{1}H NMR

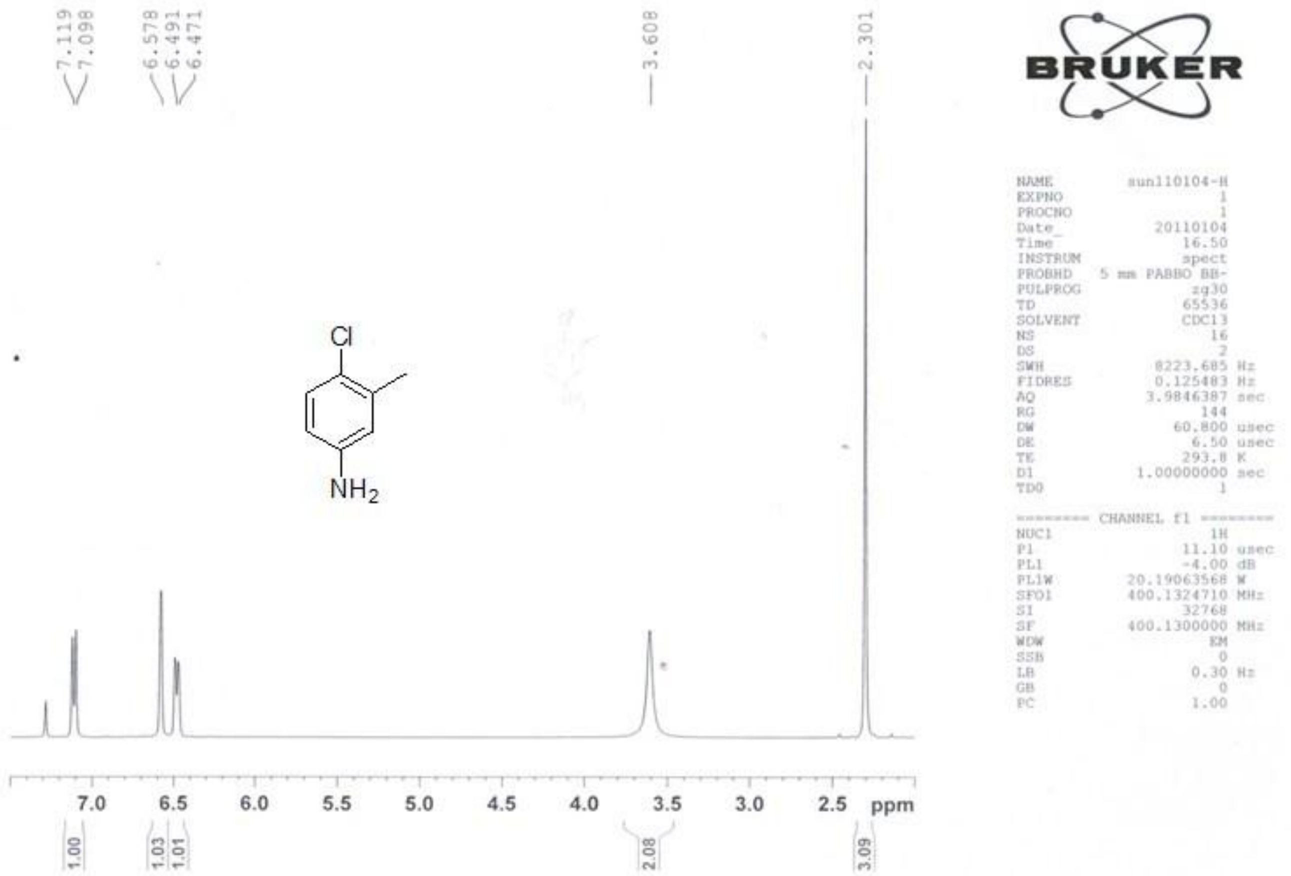

4-CHLORO-3-METHYLANILINE (**4f**): ^{13}C NMR

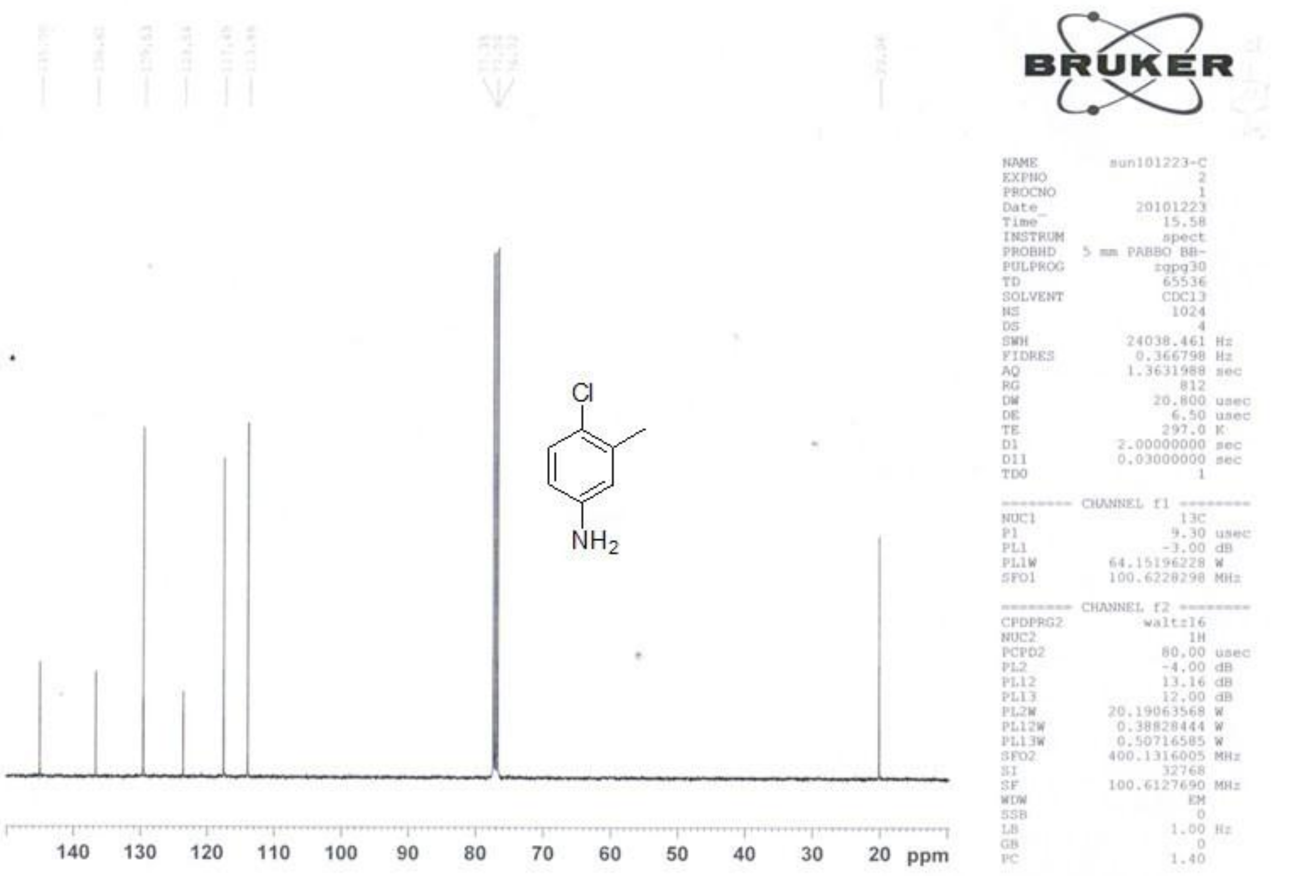

4-CHLORO-3-METHOXYANILINE (**4g**): ^{1}H NMR

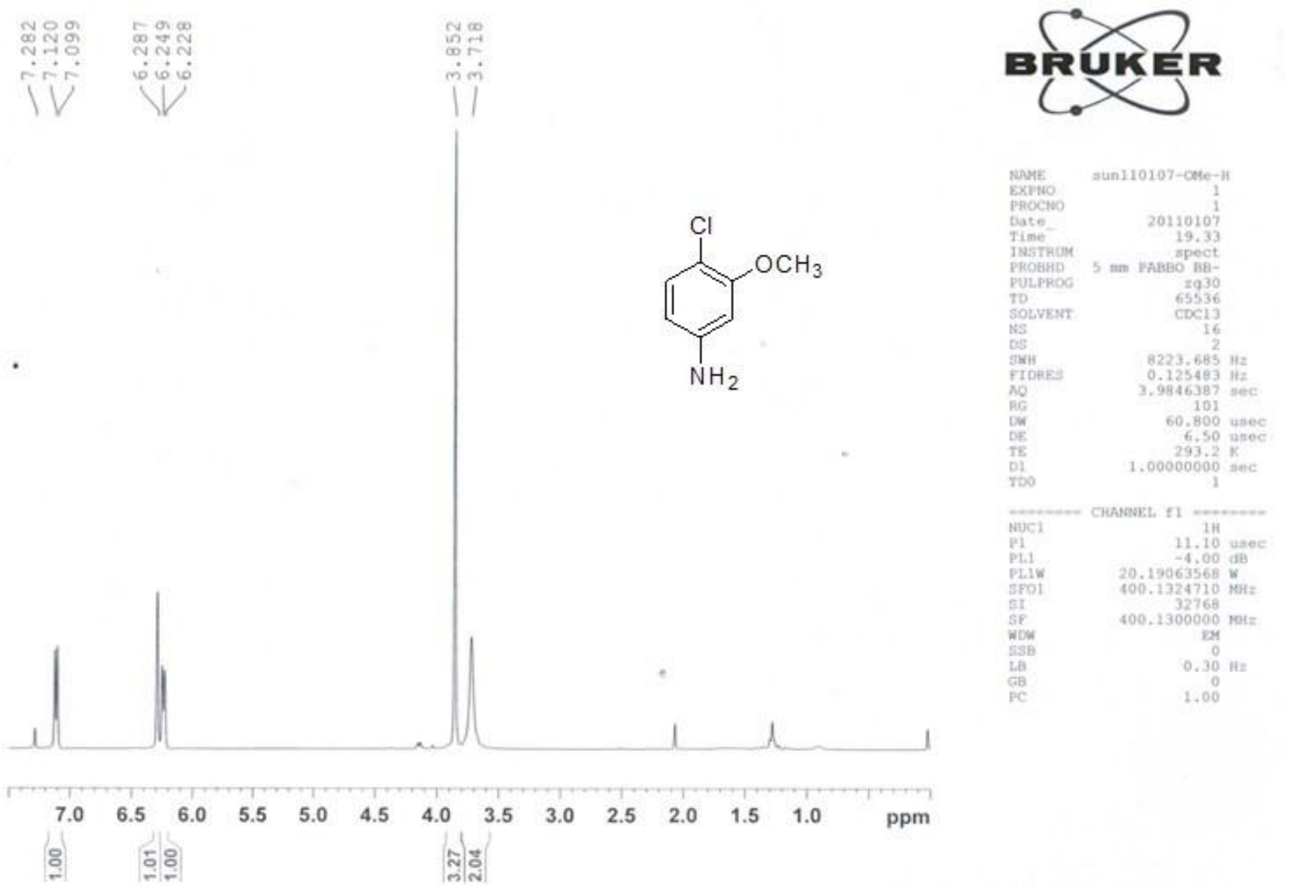

4-CHLORO-3-METHOXYANILINE (**4g**): ^{13}C NMR

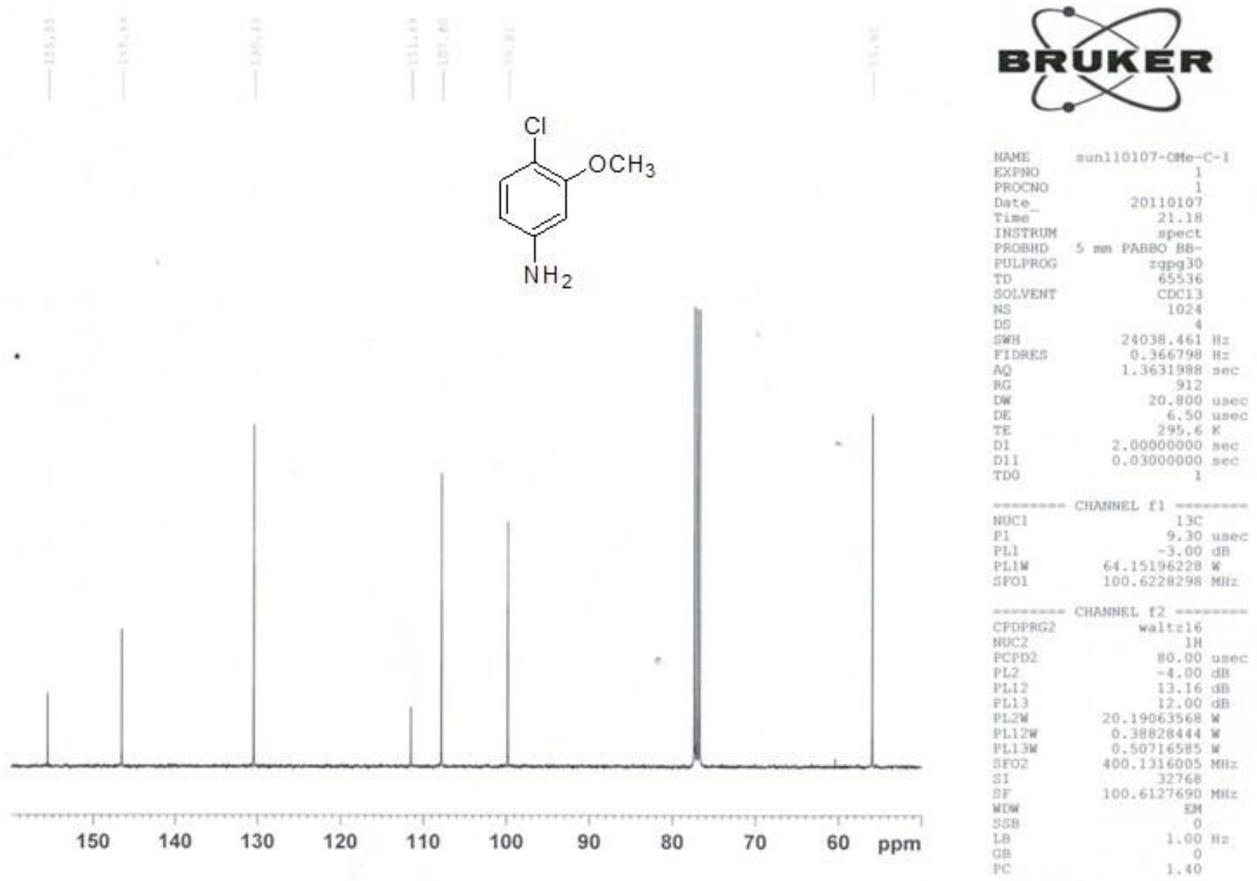

4-CHLORO-3-FLUOROANILINE (**4h**): ^{1}H NMR

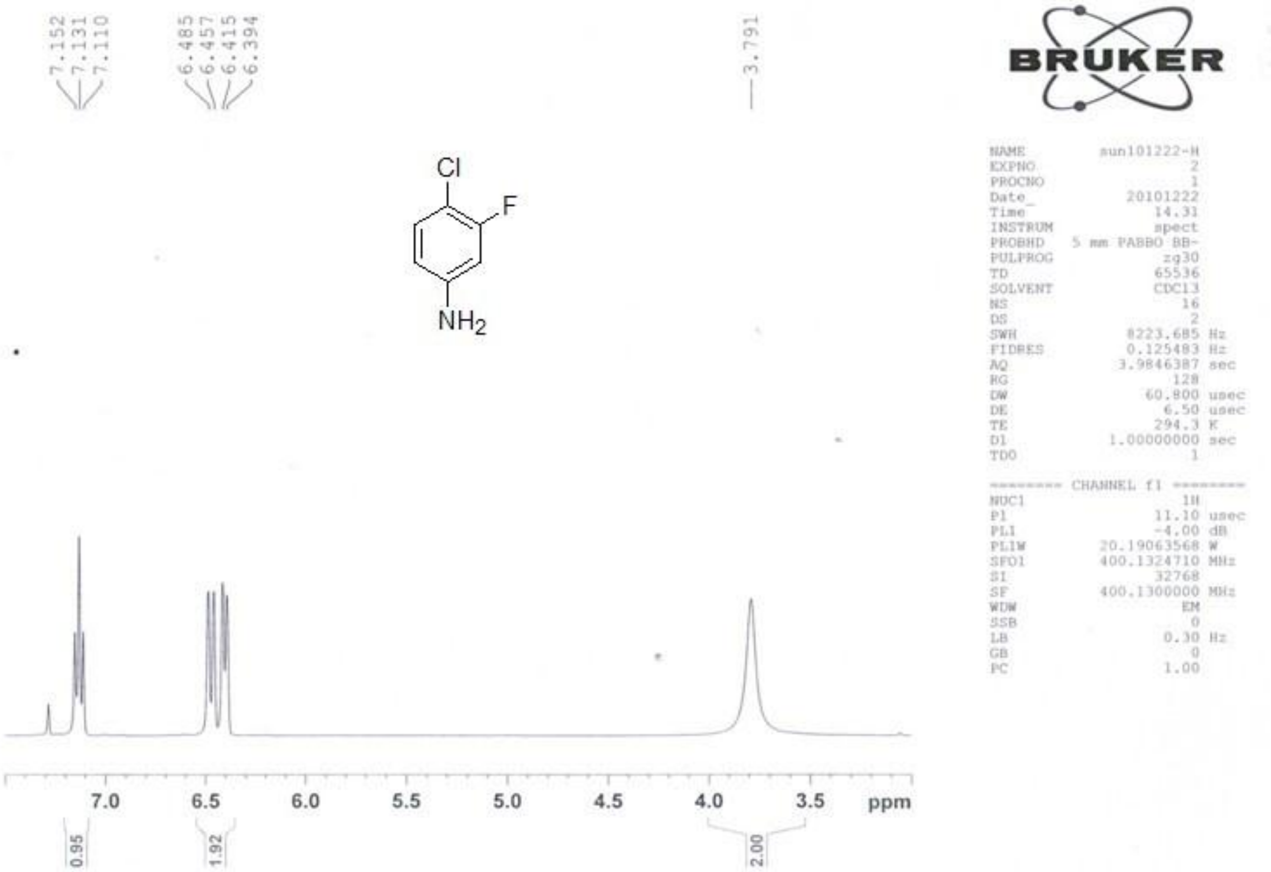

4-CHLORO-3-FLUOROANILINE (**4h**): ^{13}C NMR

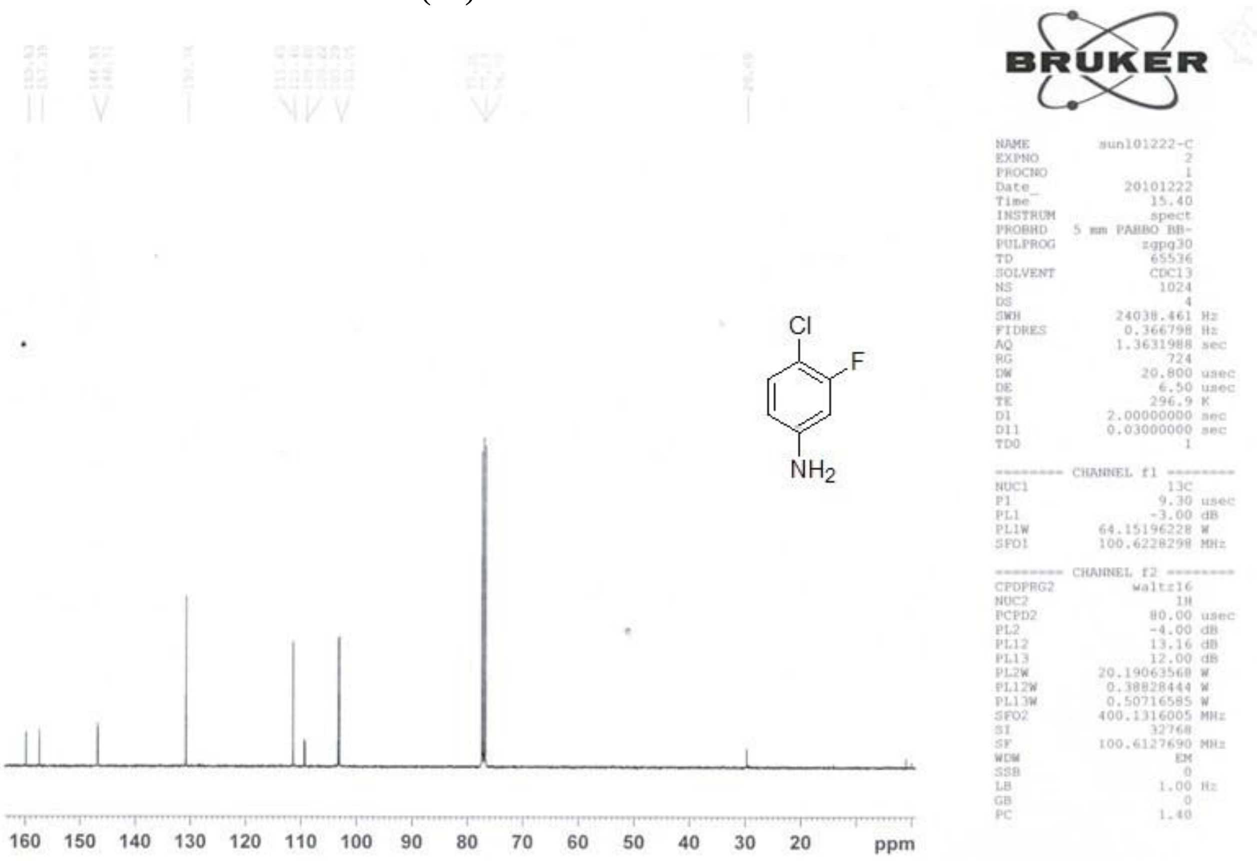

4-CHLORO-3-FLUOROANILINE (**4h**): ^{19}F NMR

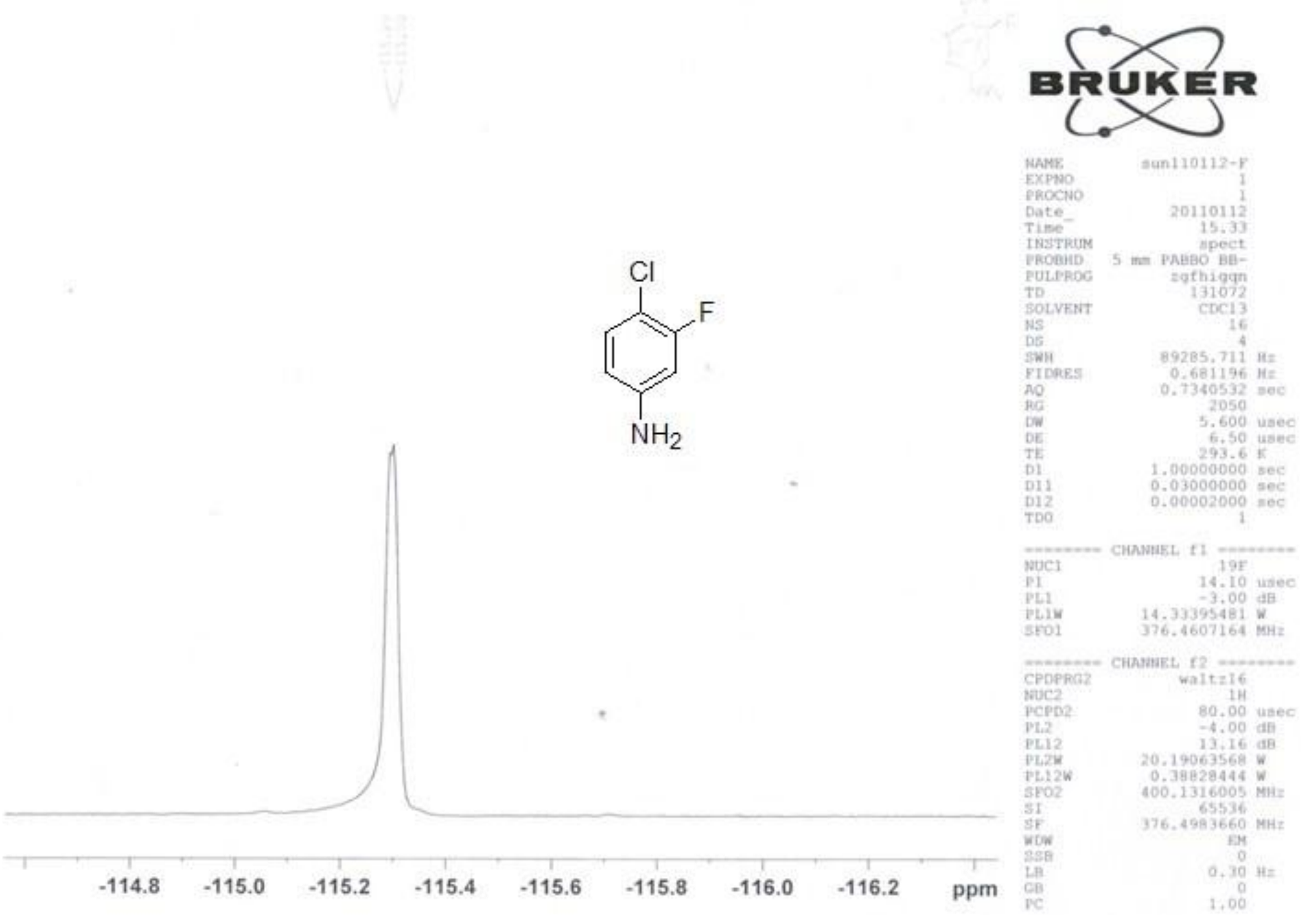

4-CHLORO-3-TRIFLUOROMETHYLANILINE (**4i**): ^{1}H NMR

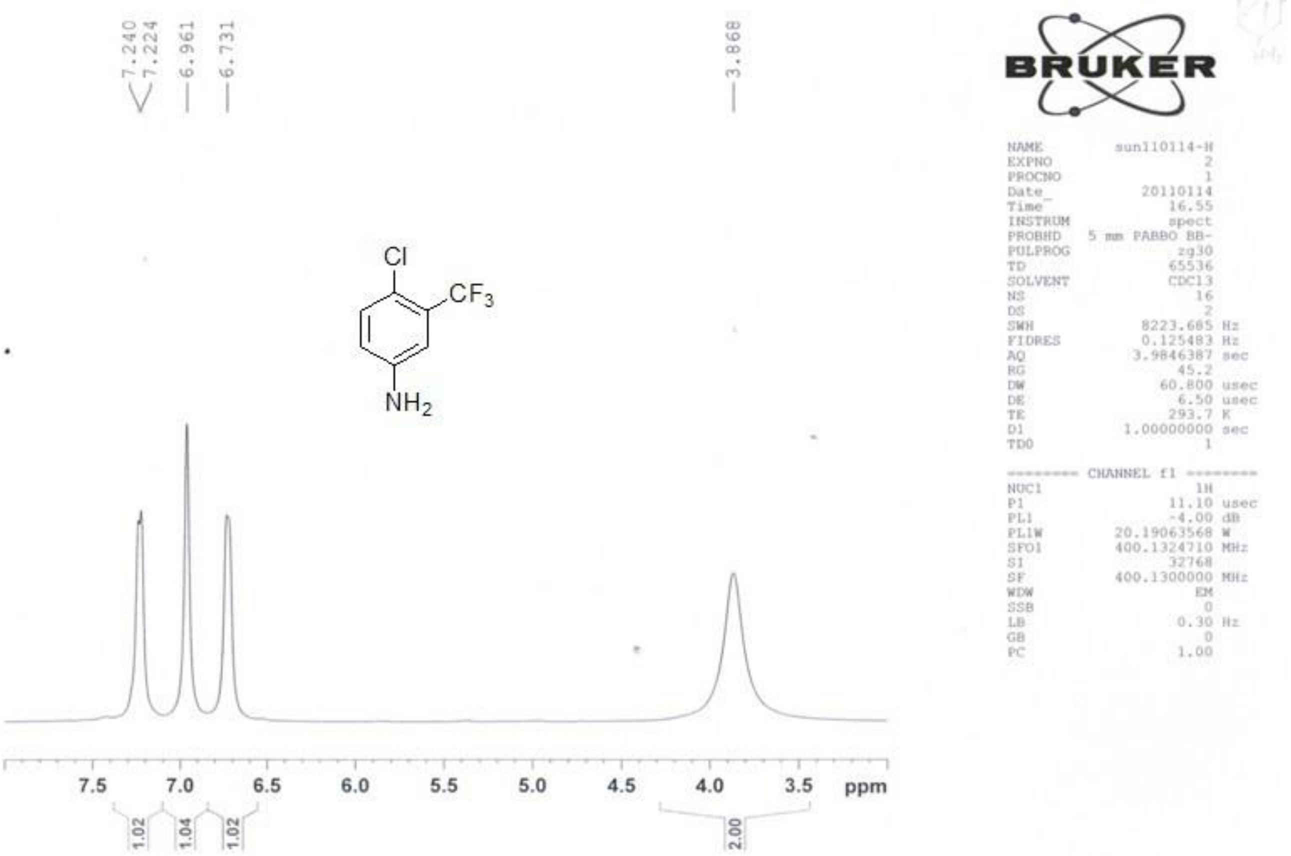

4-CHLORO-3-TRIFLUOROMETHYLANILINE (**4i**): ^{13}C NMR

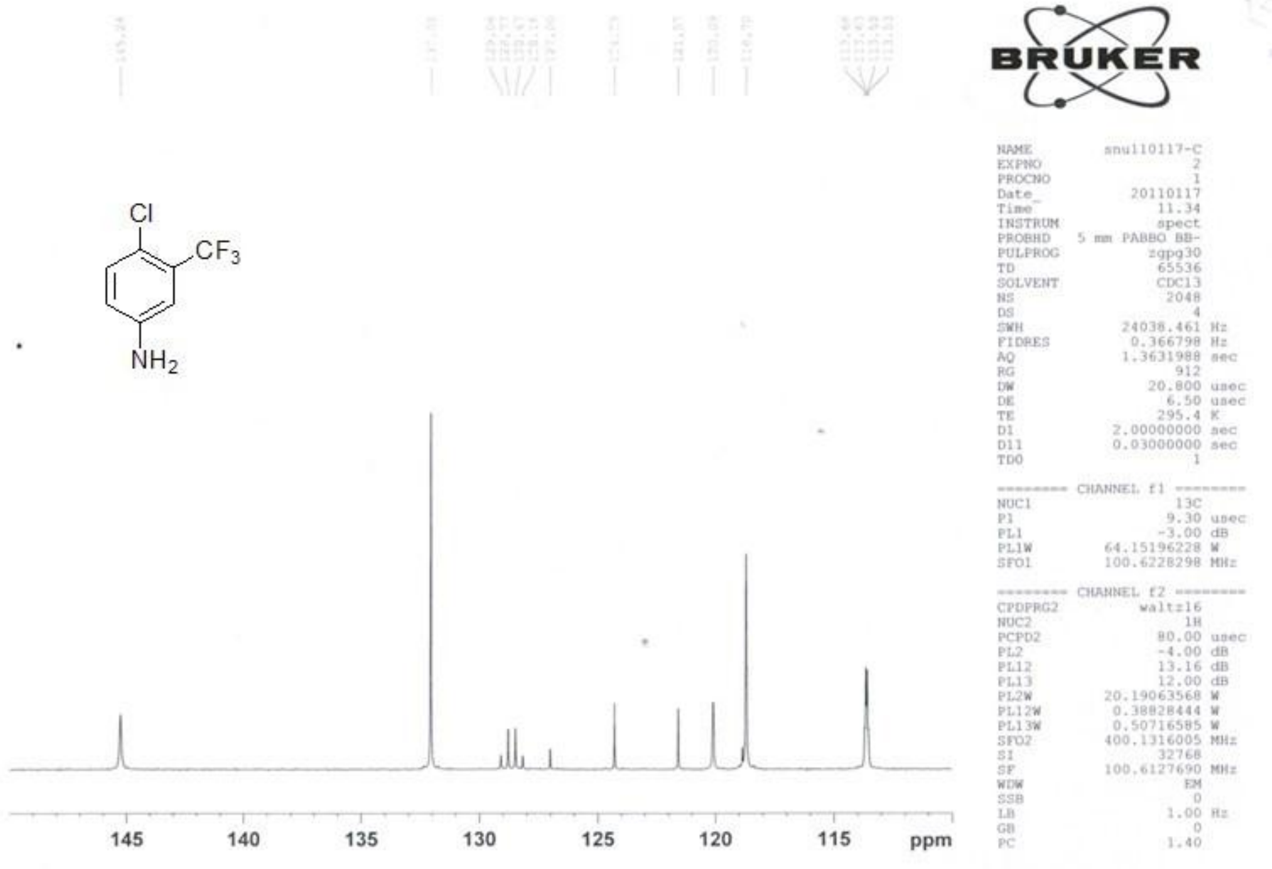

4-CHLORO-3-TRIFLUOROMETHYLANILINE (**4i**): ^{19}F NMR

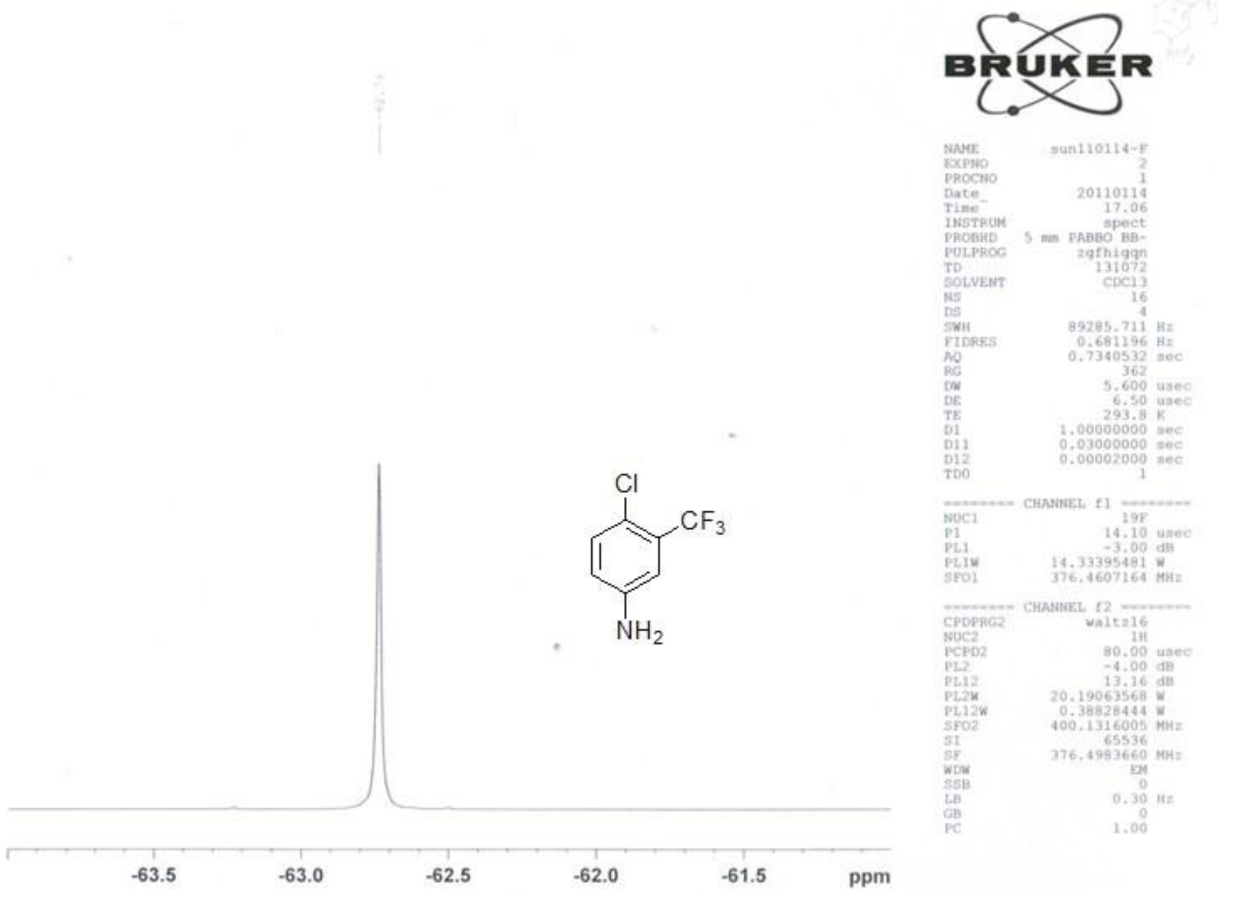

4-BROMO-2-METHYLANILINE (**5a**): ^{1}H NMR

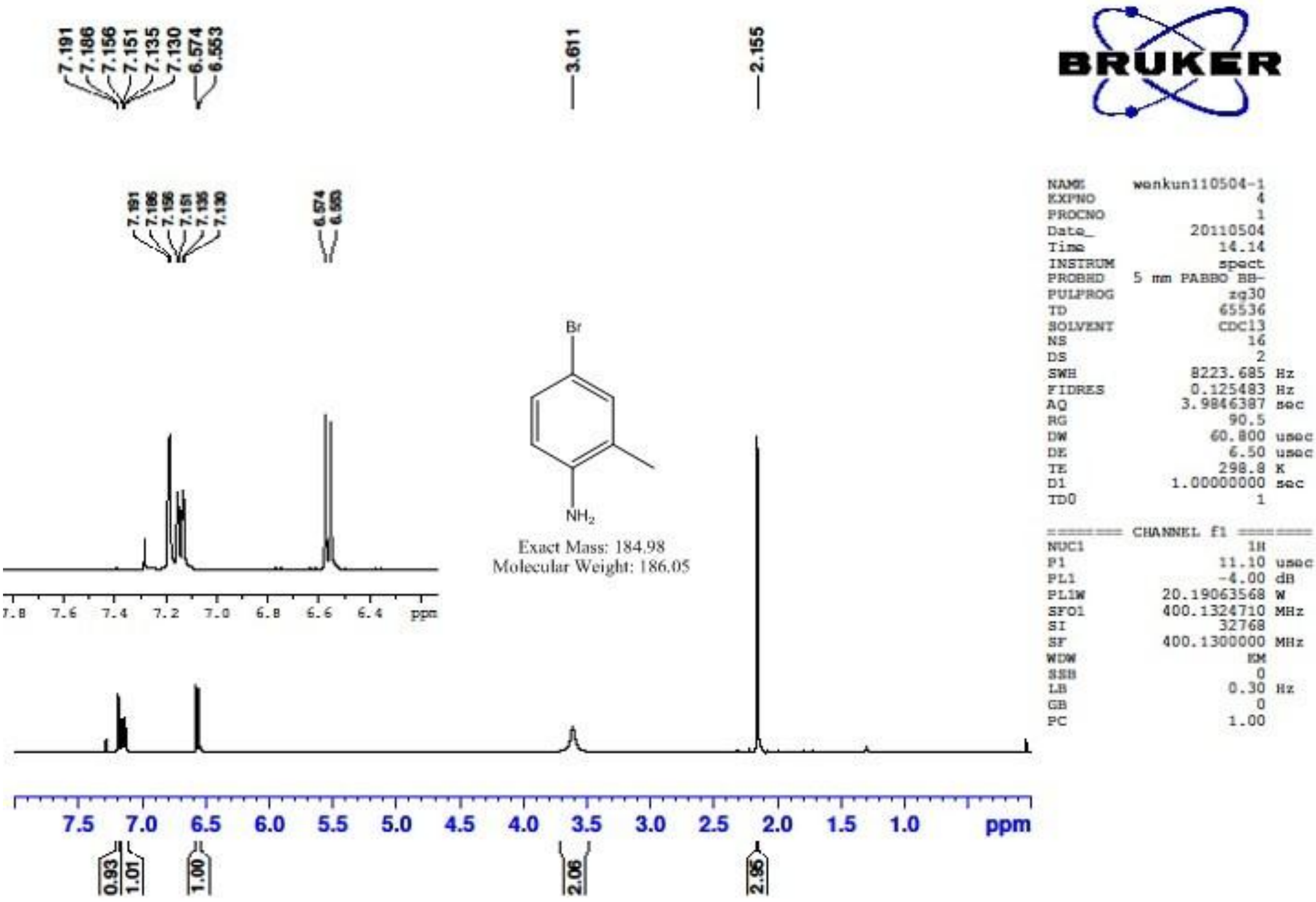

4-BROMO-2-METHYLANILINE (**5a**): ^{13}C NMR

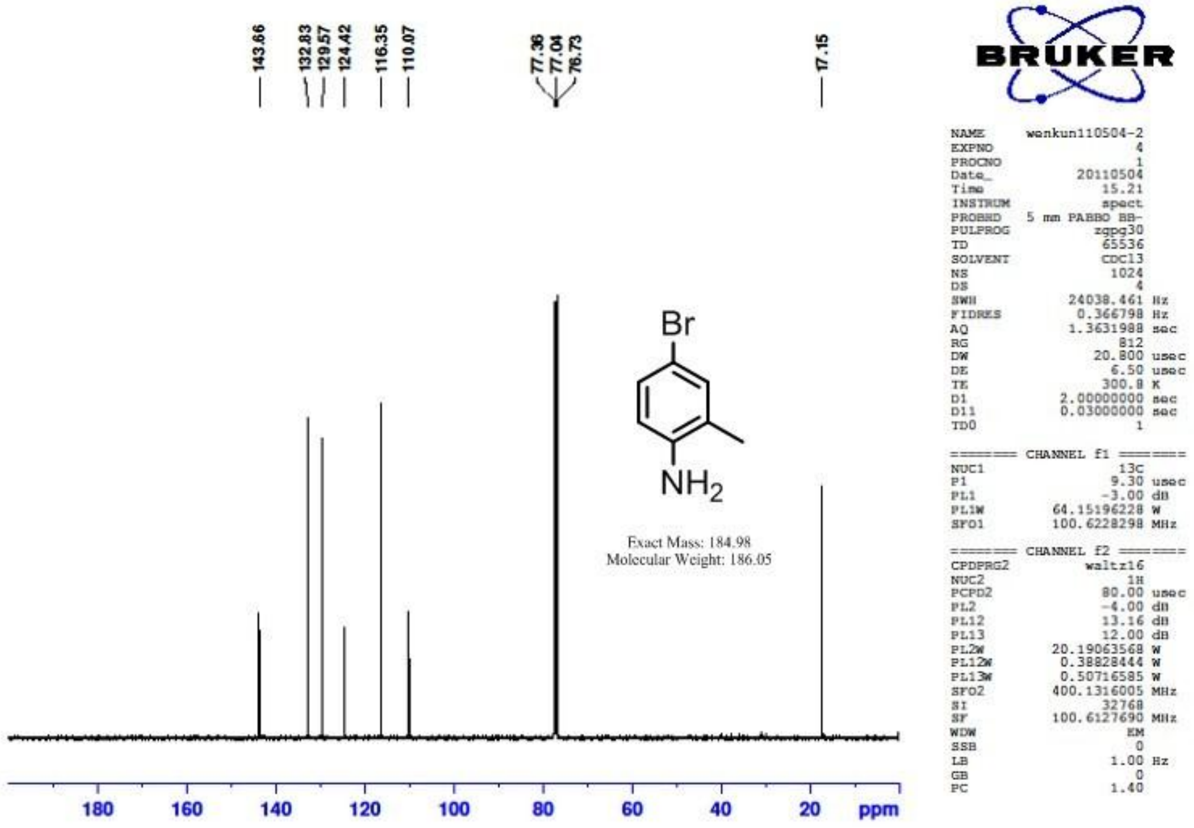

4-BROMO-2-METHOXYANILINE (**5b**): ^{1}H NMR

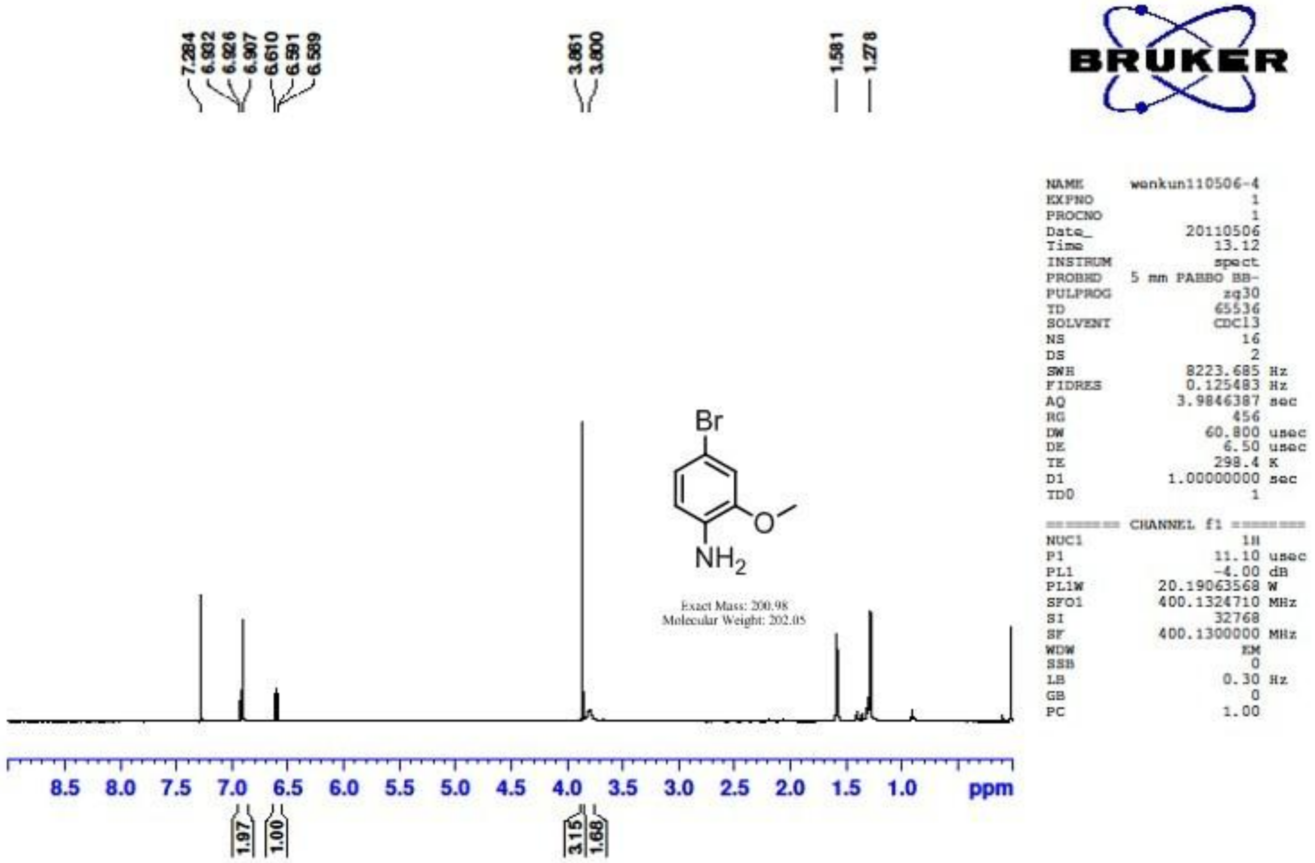

4-BROMO-2-METHOXYANILINE (**5b**): ^{13}C NMR

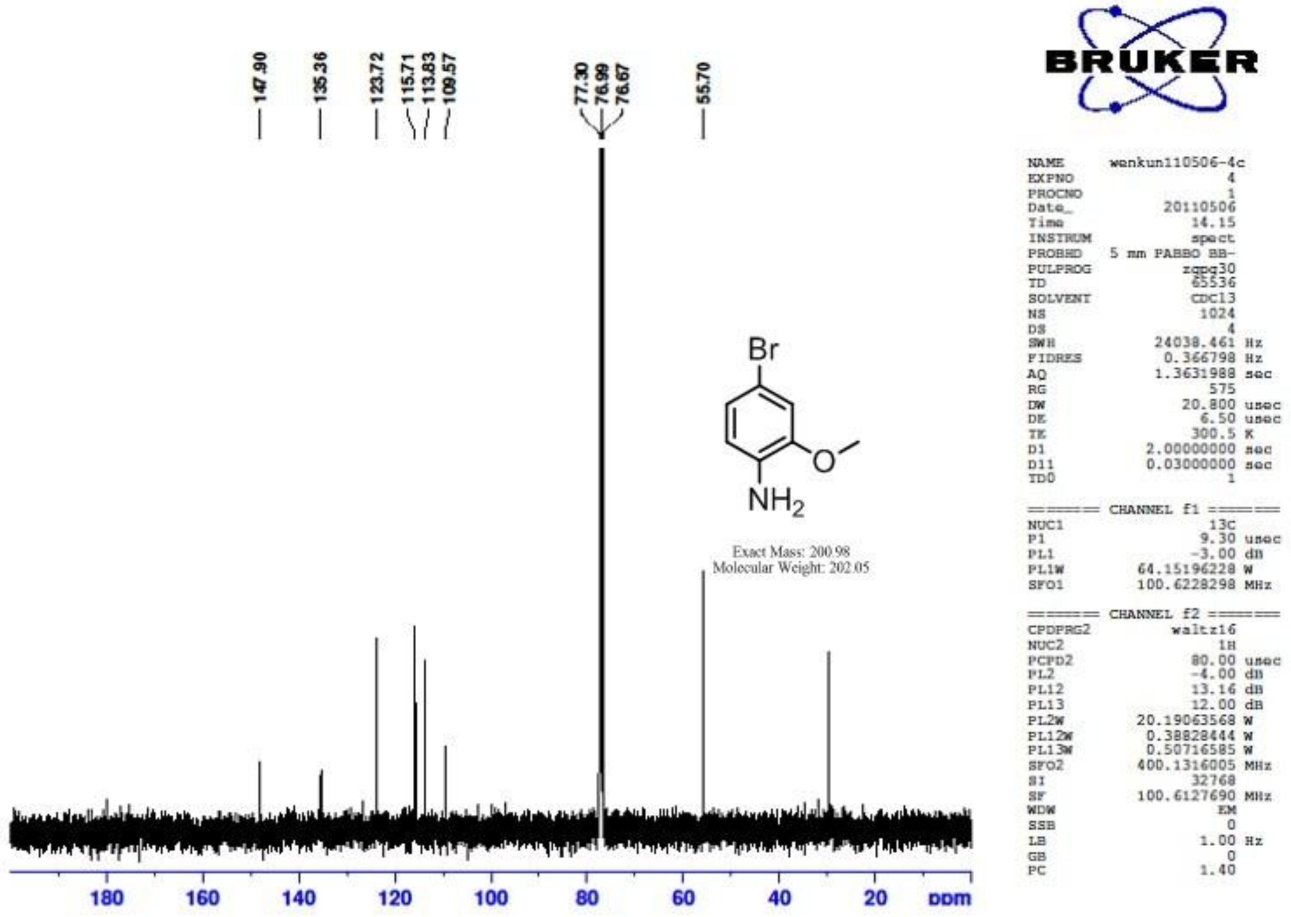

4-BROMO-2-FLUOROANILINE (**5c**): ^{1}H NMR

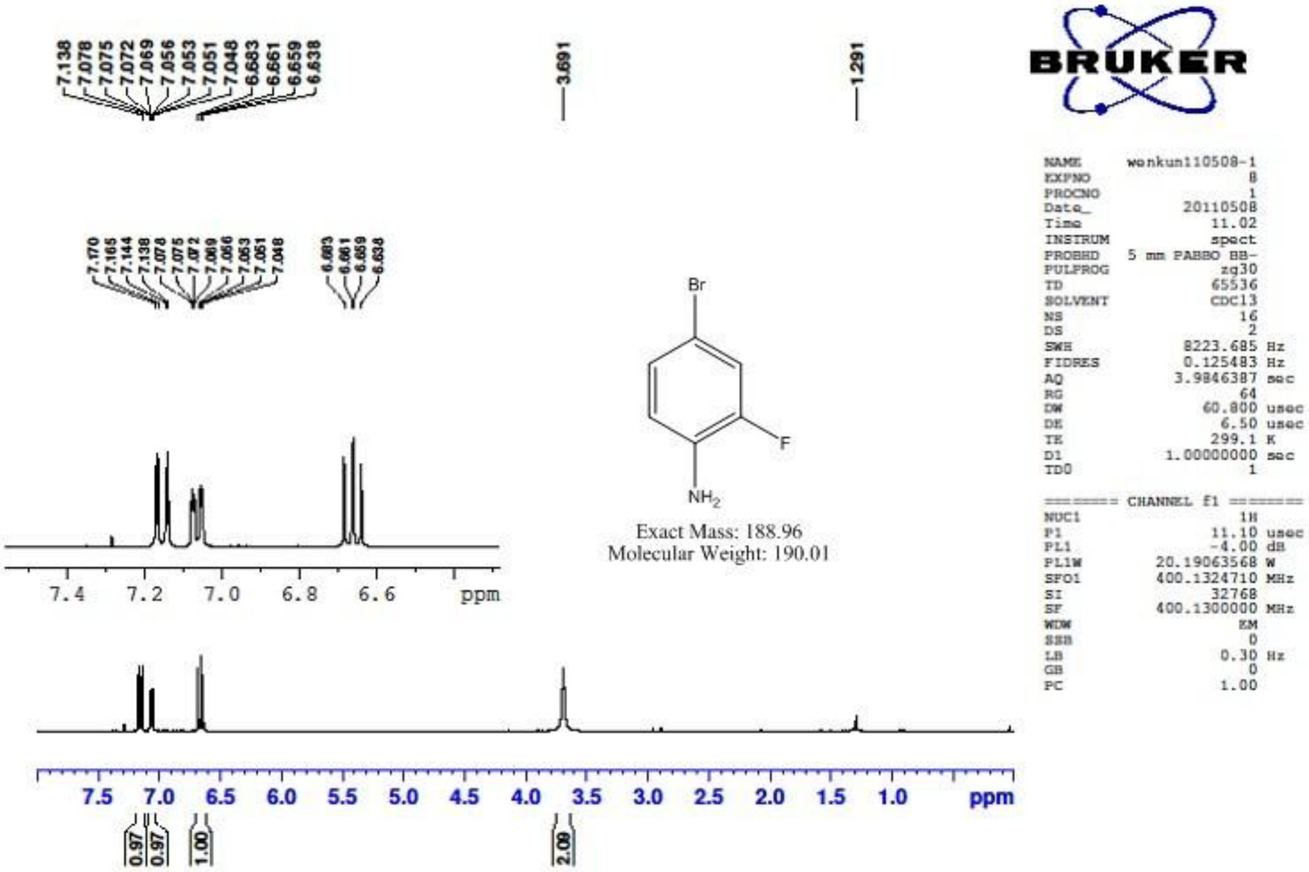

4-BROMO-2-FLUOROANILINE (**5c**): ^{13}C NMR

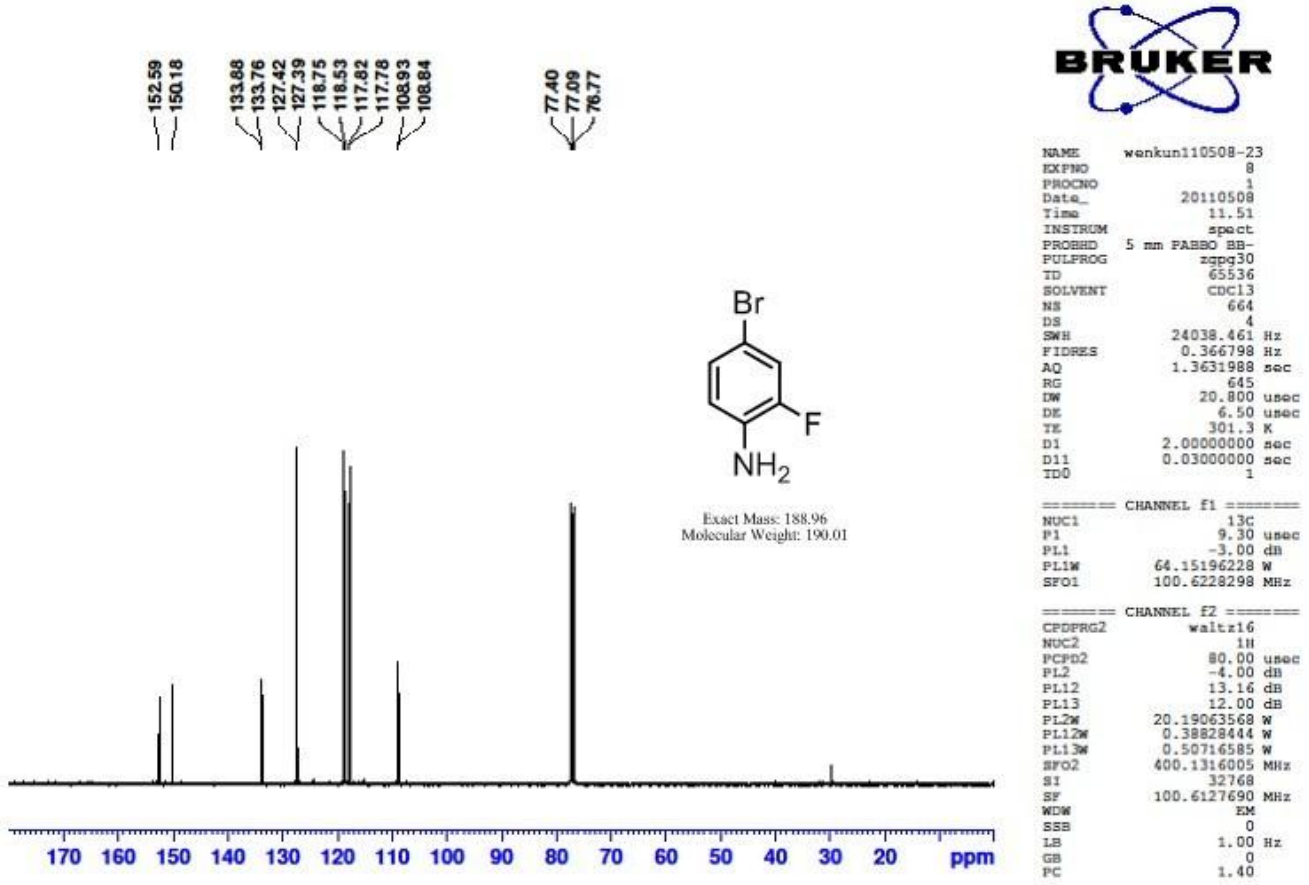

4-BROMO-2-FLUOROANILINE (**5c**): ^{19}F NMR

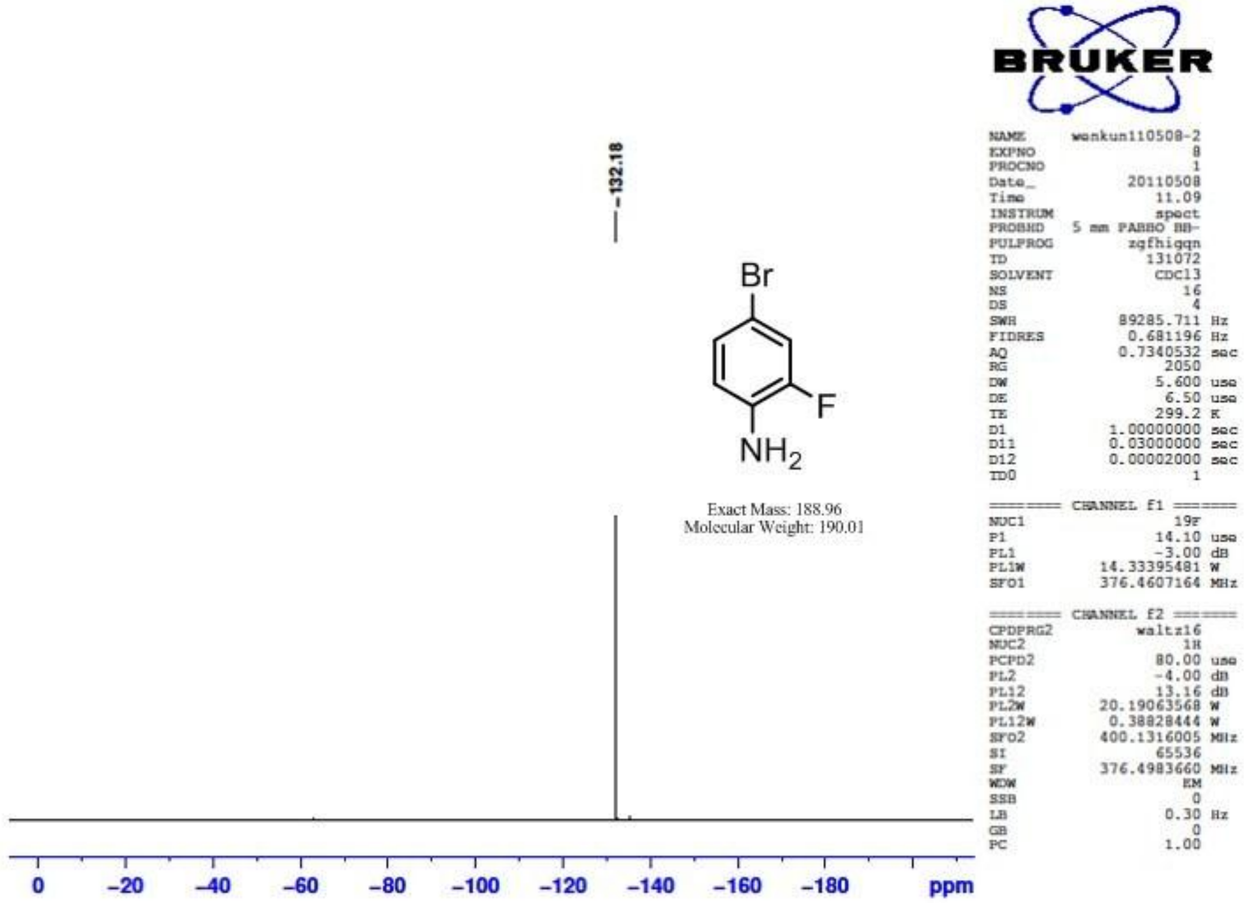

4-BROMO-2-TRIFLUOROMETHYLANILINE (**5d**): ^{1}H NMR

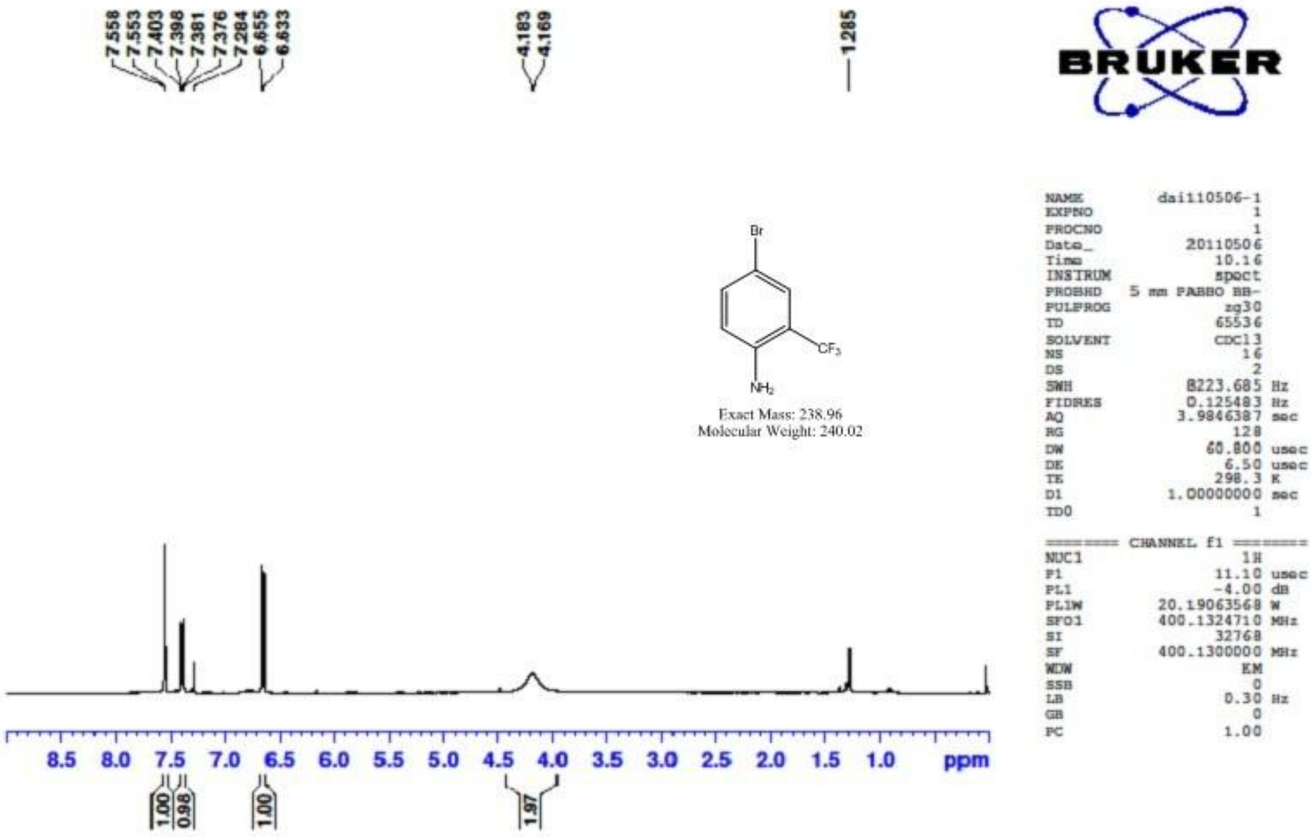

4-BROMO-2-TRIFLUOROMETHYLANILINE (**5d**): ^{13}C NMR

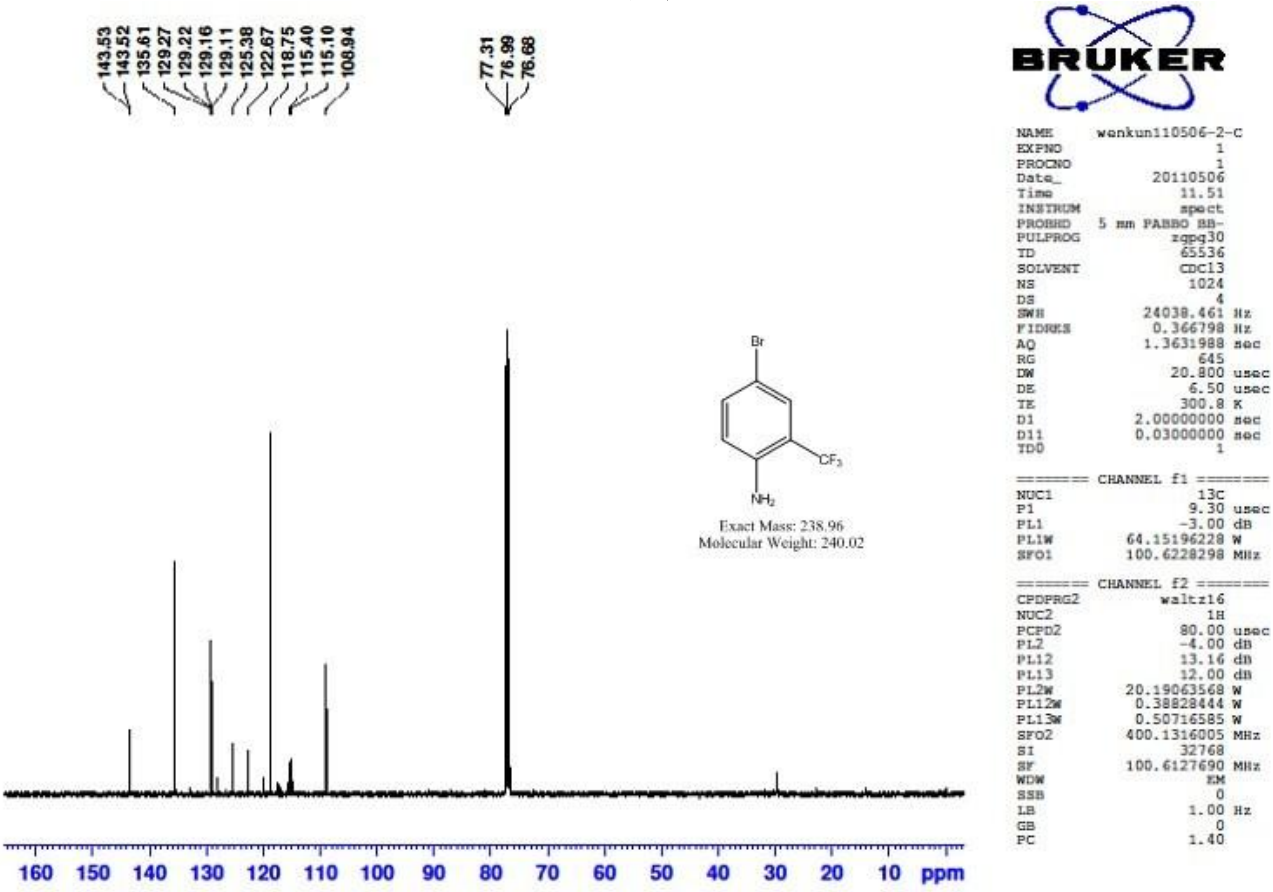

4-BROMO-2-TRIFLUOROMETHYLANILINE (**5d**): ^{19}F NMR

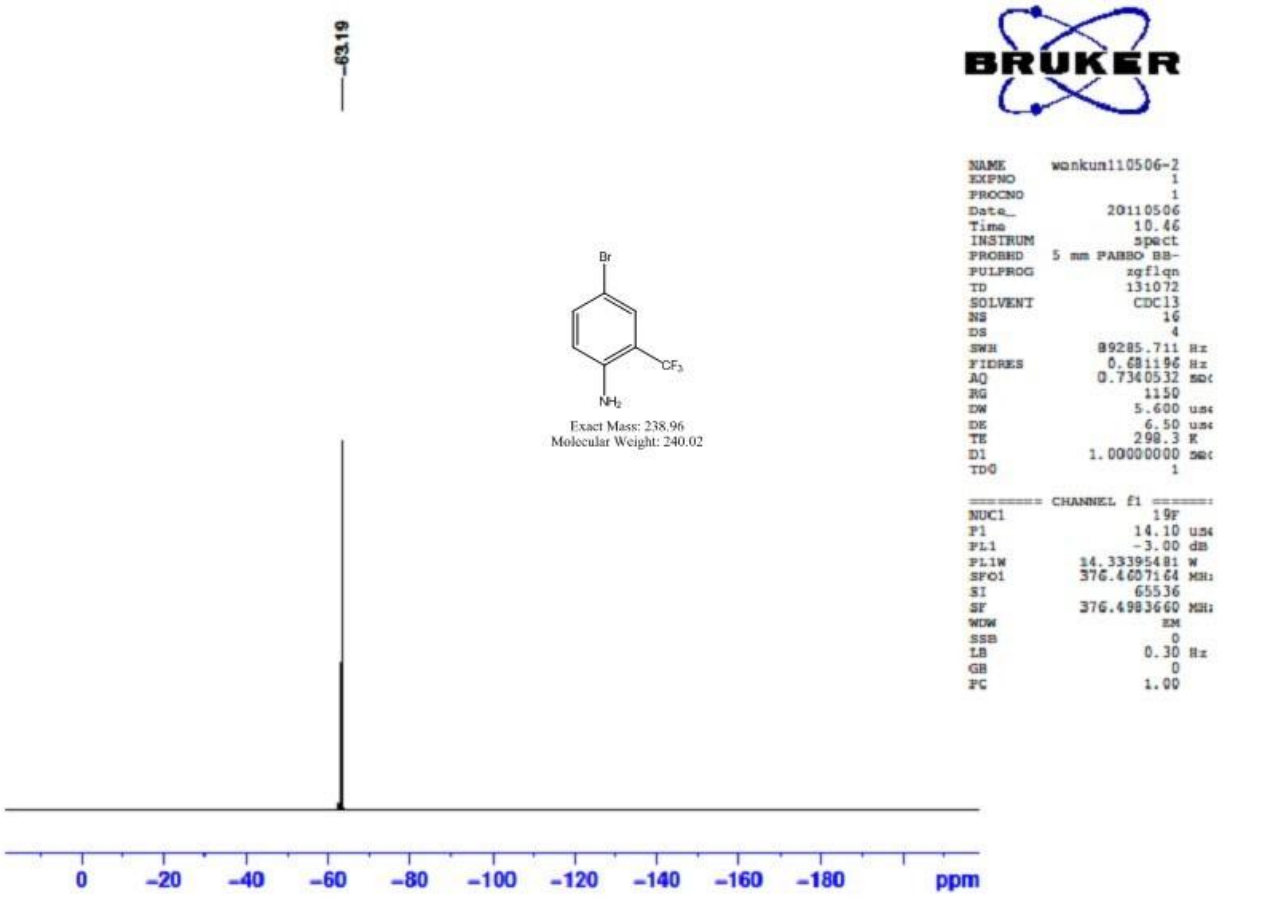

4-BROMO-2-NITROANILINE (**5e**): ^{1}H NMR

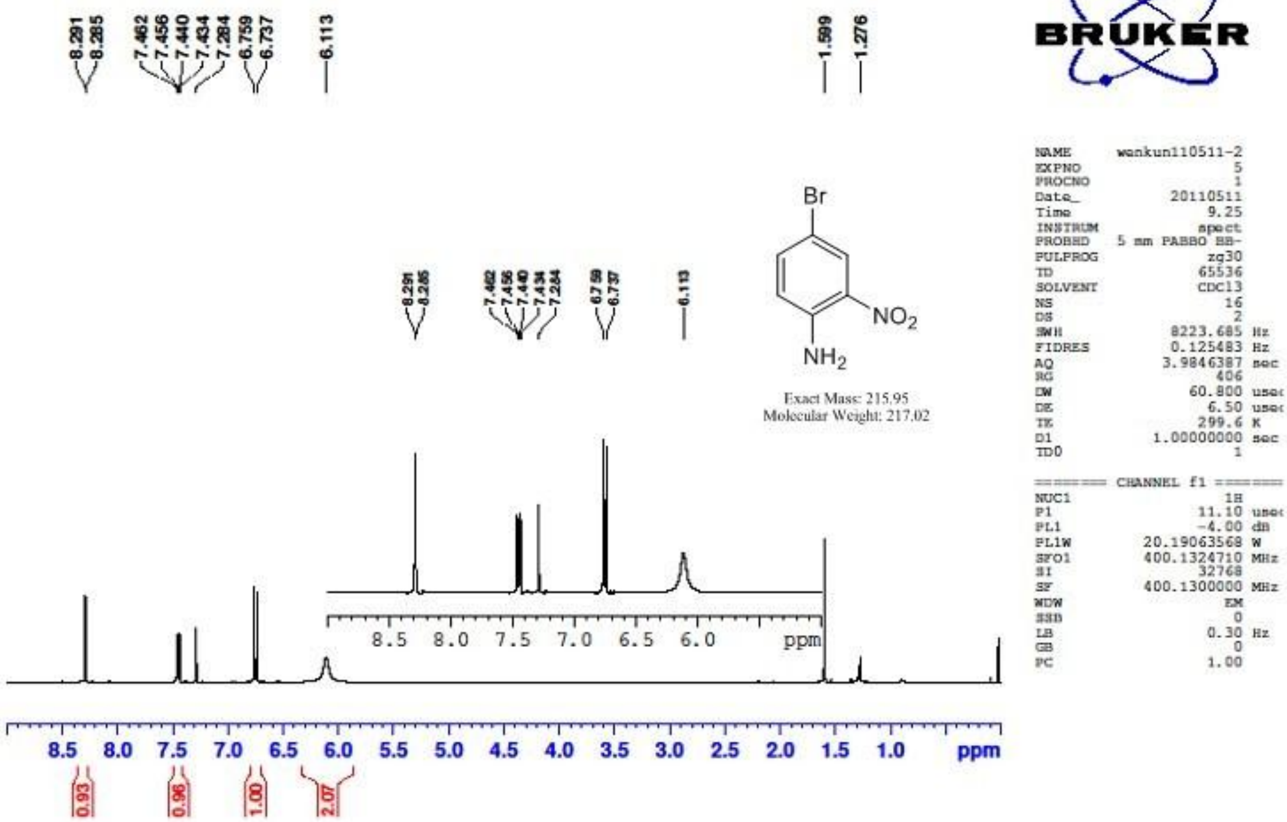

4-BROMO-2-NITROANILINE (**5e**): ^{13}C NMR

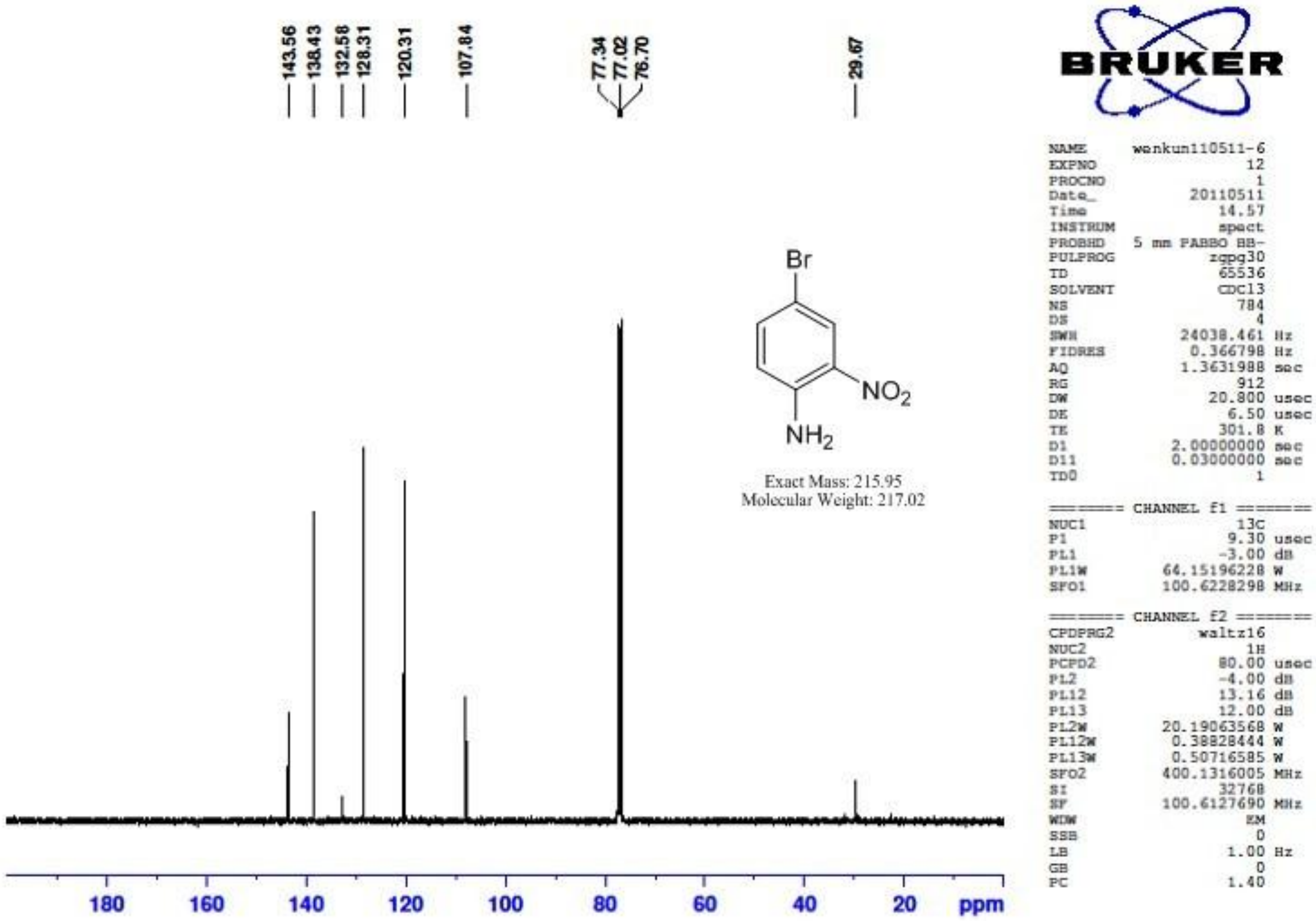

4-BROMO-3-METHYLANILINE (**5f**): ^{1}H NMR

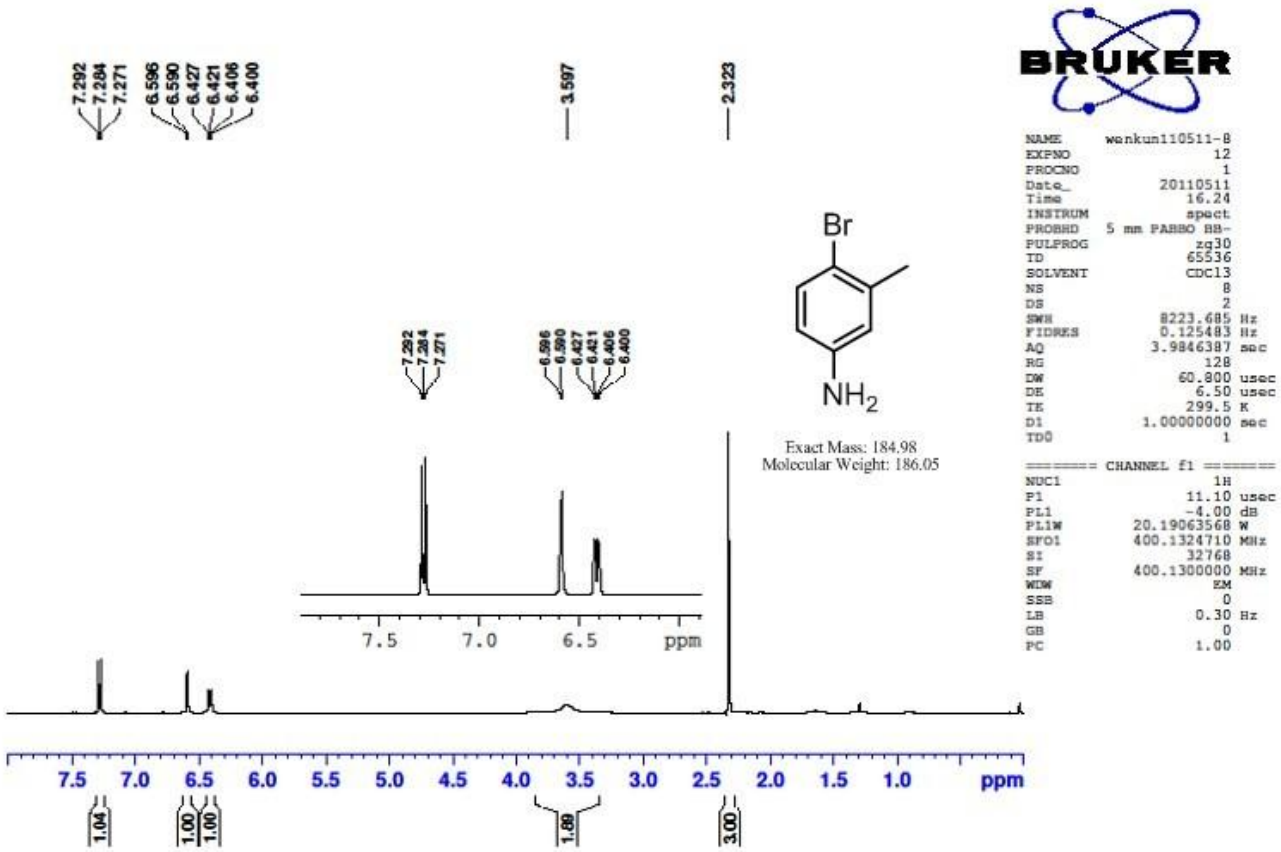

4-BROMO-3-METHYLANILINE (**5f**): ^{13}C NMR

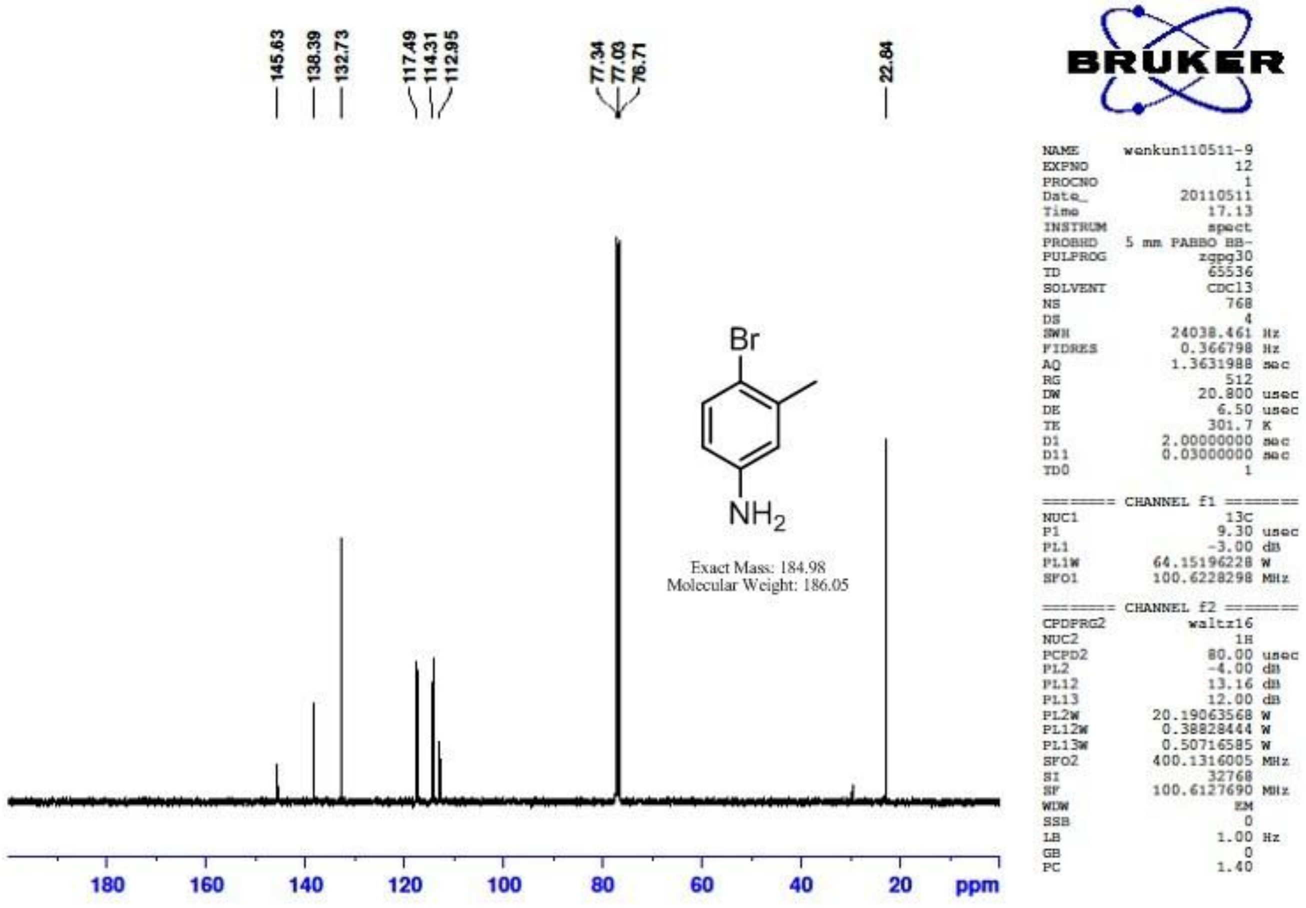

4-BROMO-3-METHOXYANILINE (**5g**): ^{1}H NMR

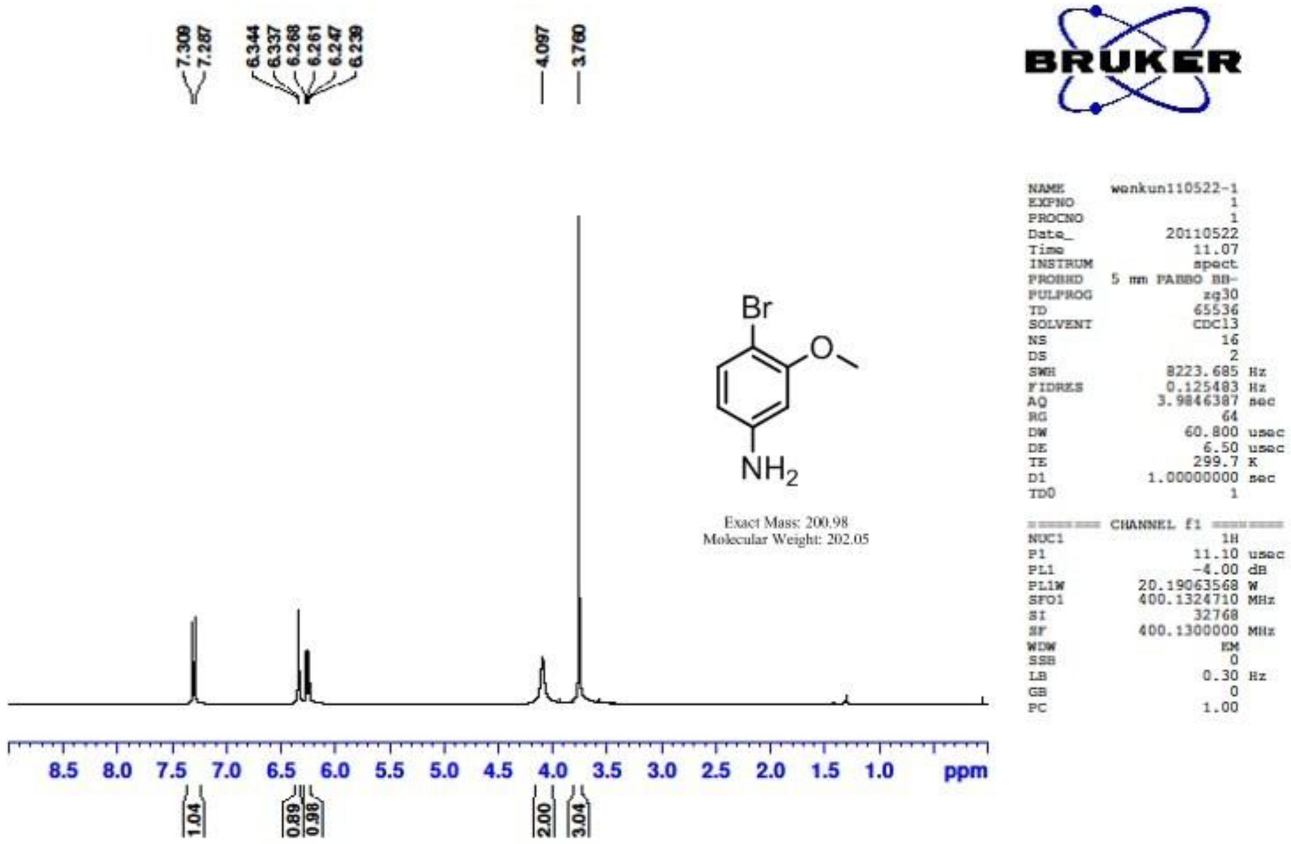

4-BROMO-3-METHOXYANILINE (**5g**): ^{13}C NMR

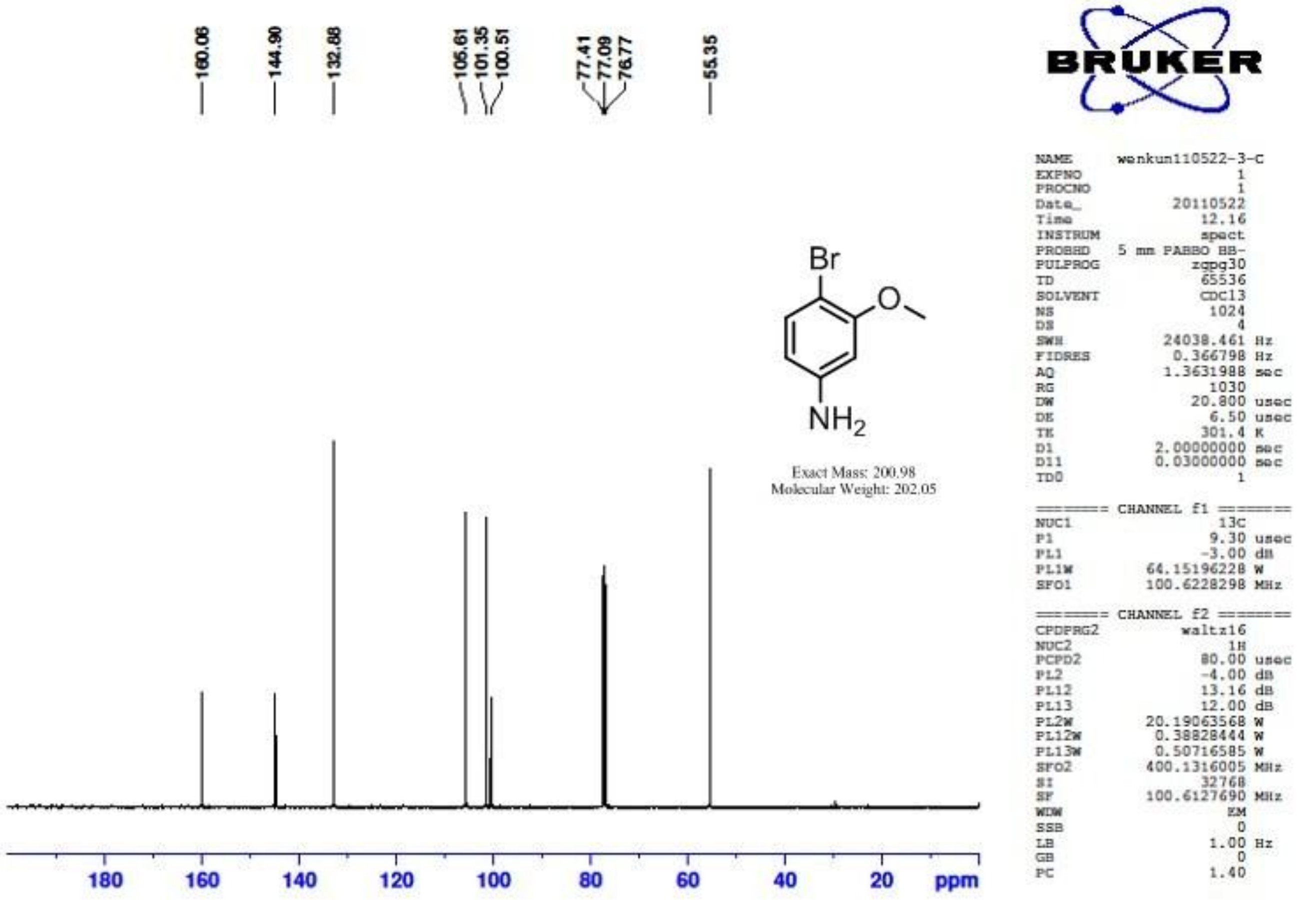

4-BROMO-3-FLUOROANILINE (**5h**): ^{1}H NMR

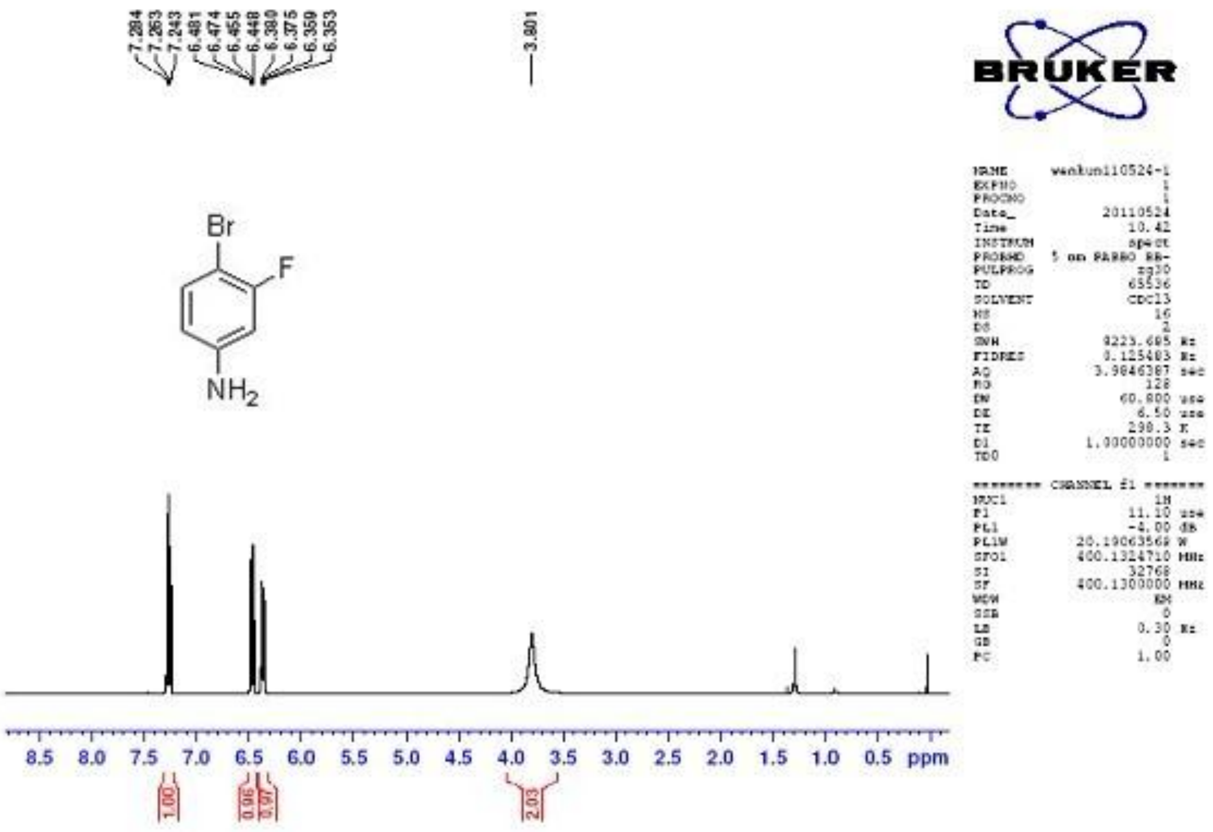

4-BROMO-3-FLUOROANILINE (**5h**): ^{13}C NMR

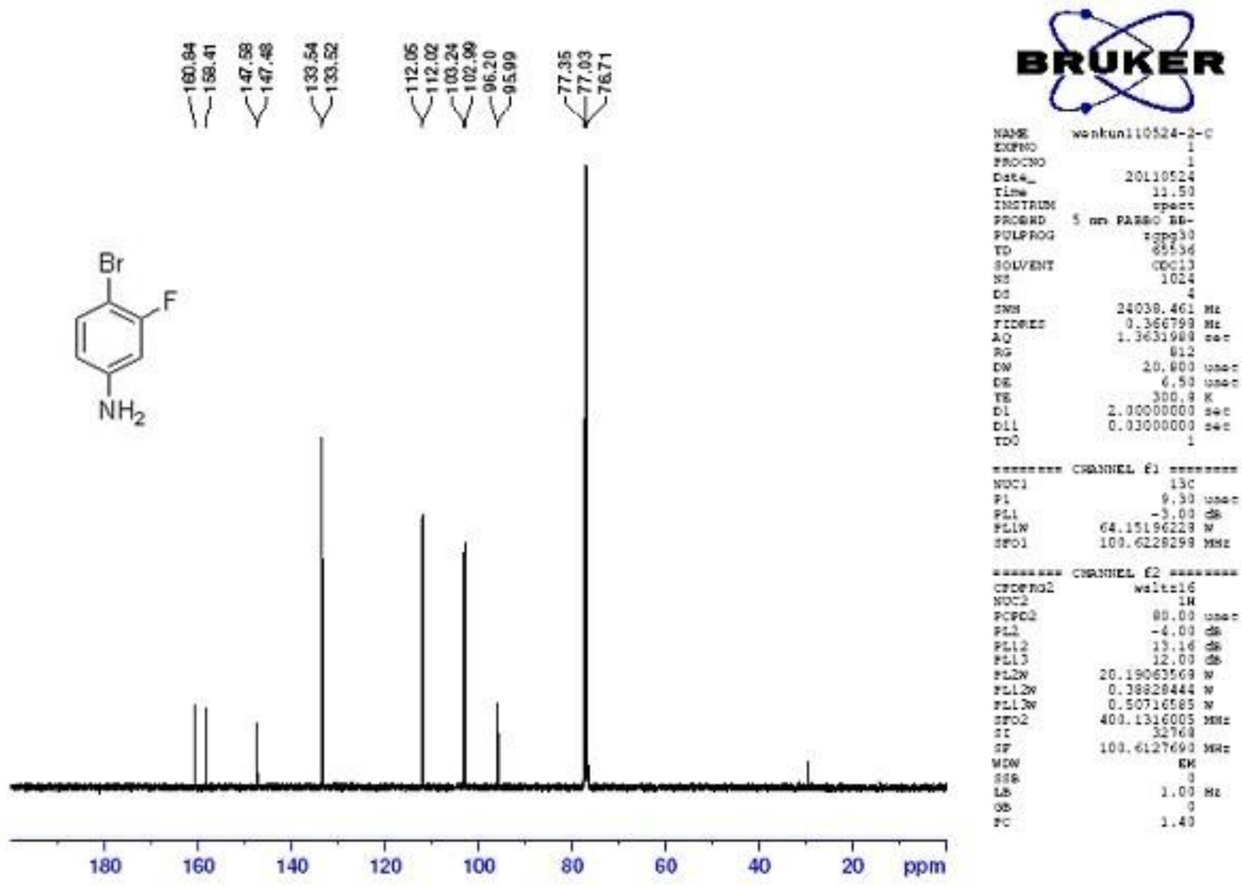

4-BROMO-3-FLUOROANILINE (**5h**): ^{19}F NMR

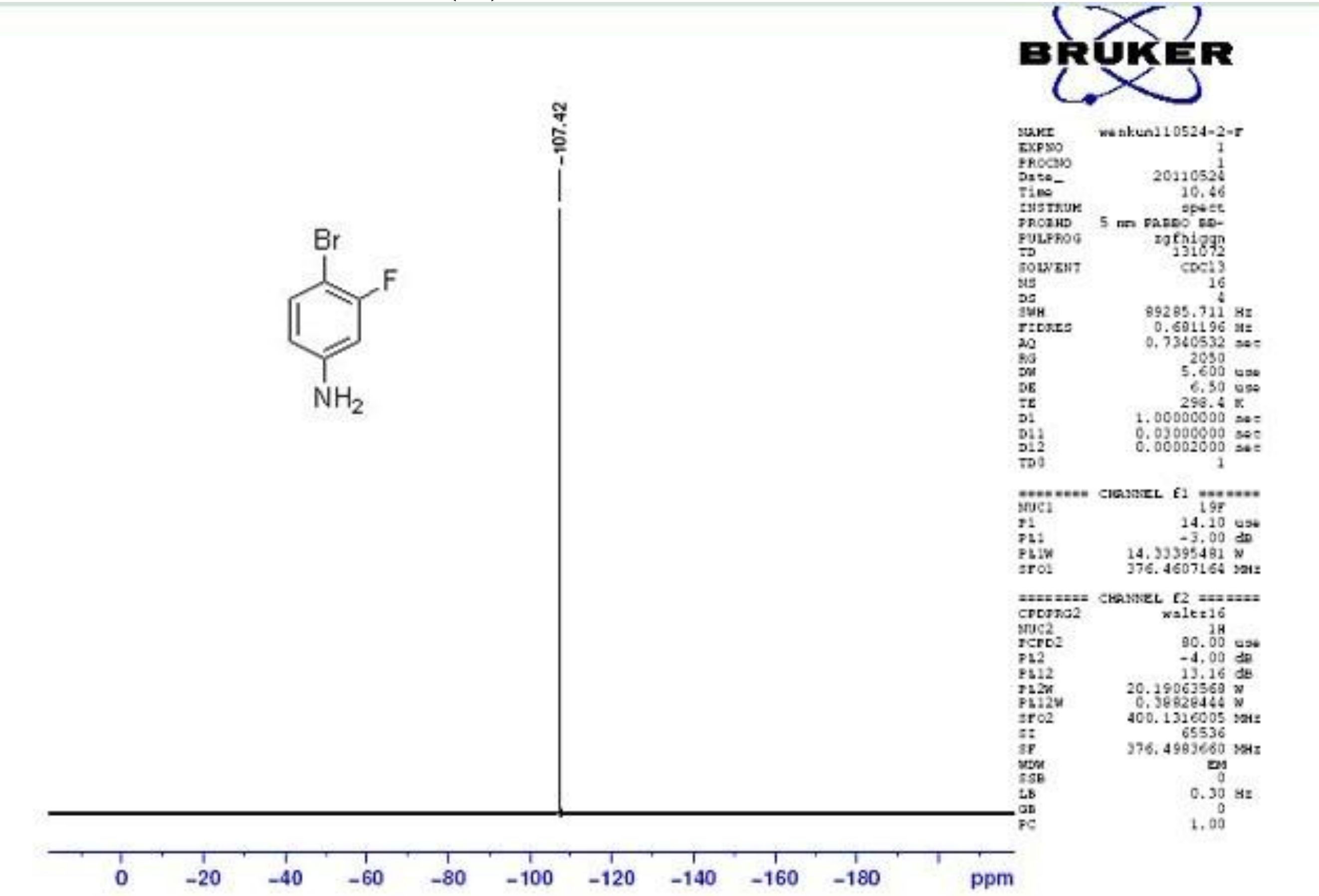

4-BROMO-3-TRIFLUOROMETHYLANILINE (**5i**): ^{1}H NMR

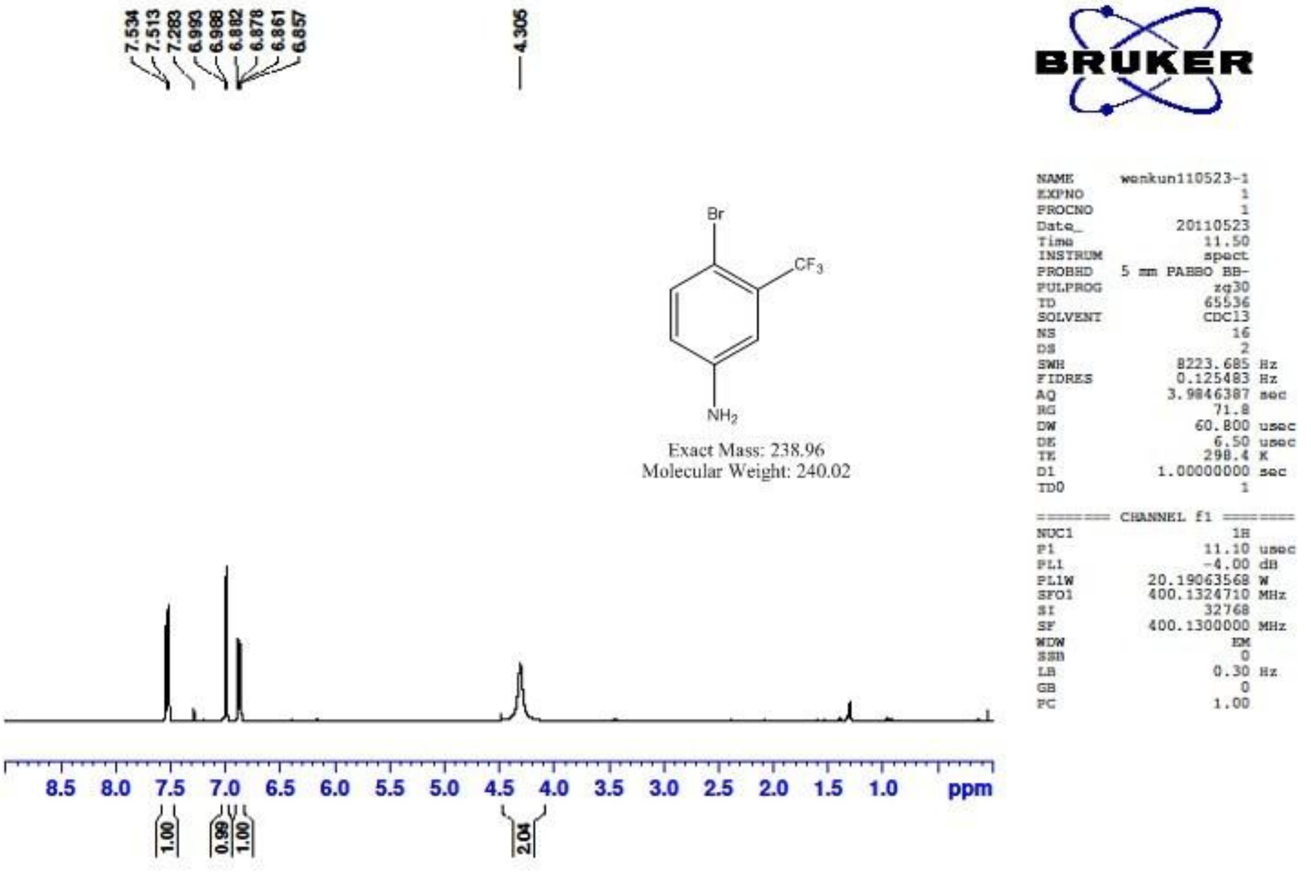

4-BROMO-3-TRIFLUOROMETHYLANILINE (**5i**): ^{13}C NMR

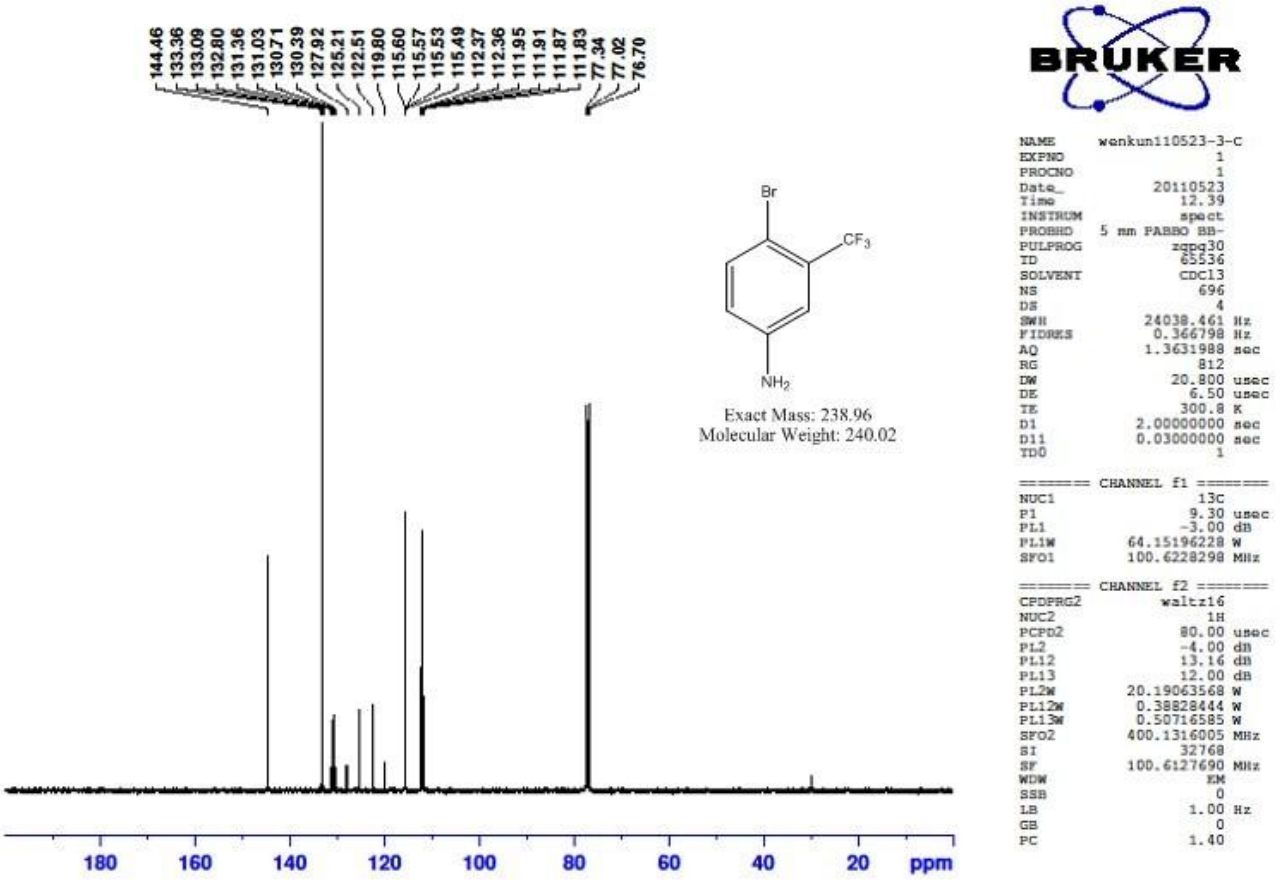

4-BROMO-3-TRIFLUOROMETHYLANILINE (**5i**): ^{19}F NMR

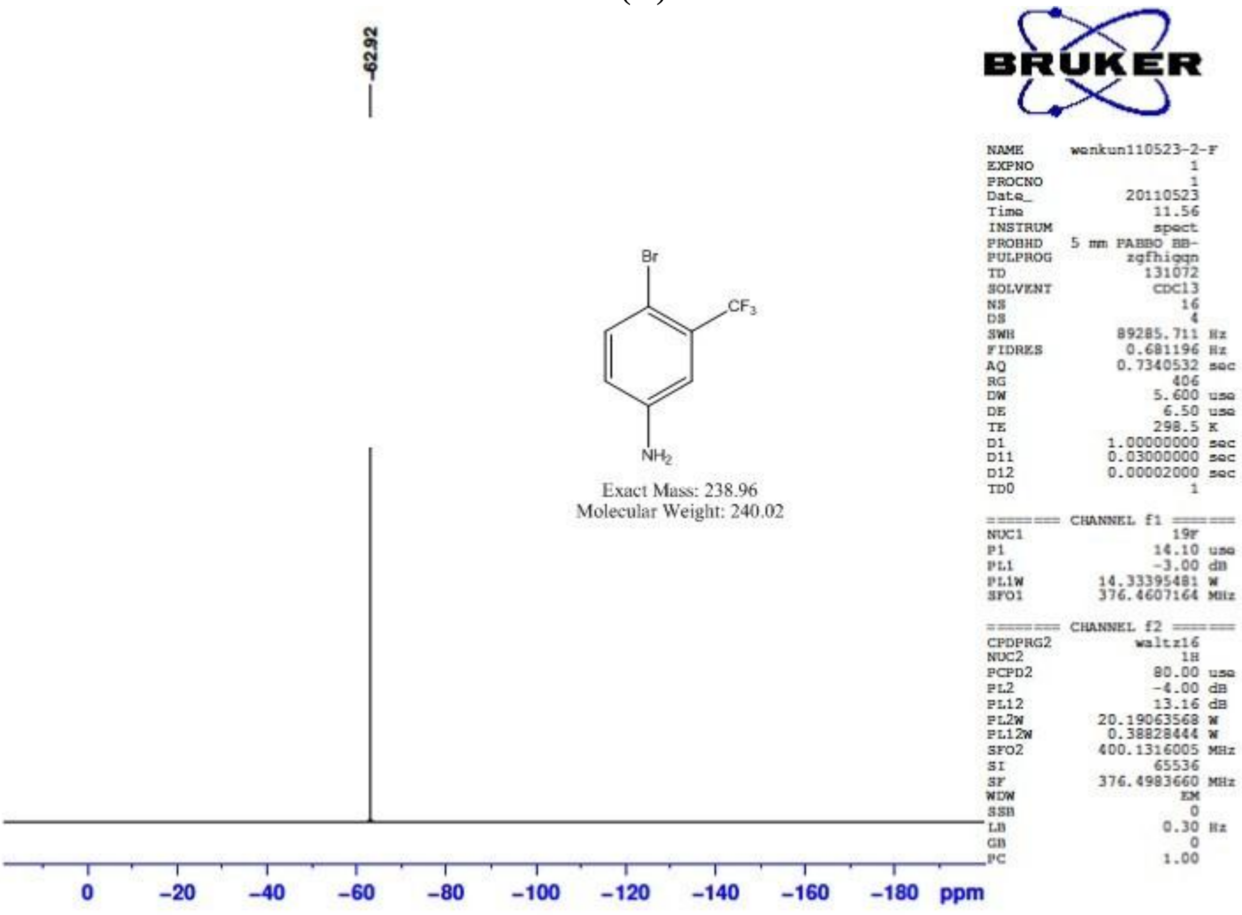

Supplement: File 2 — NMR spectra of all compounds. [file Beilstein_J_Org_Chem-08-744-s002.pdf]
